# Cleavage of DFNA5 by caspase-3 during apoptosis mediates progression to secondary necrotic/pyroptotic cell death

**DOI:** 10.1038/ncomms14128

**Published:** 2017-01-03

**Authors:** Corey Rogers, Teresa Fernandes-Alnemri, Lindsey Mayes, Diana Alnemri, Gino Cingolani, Emad S. Alnemri

**Affiliations:** 1 Department of Biochemistry and Molecular Biology, Kimmel Cancer Center, Thomas Jefferson University, Philadelphia, Pennsylvania 19107, USA; 2 Schreyer Honors College, Pennsylvania State University, 10 E College Ave, University Park, Pennsylvania 16802, USA

## Abstract

Apoptosis is a genetically regulated cell suicide programme mediated by activation of the effector caspases 3, 6 and 7. If apoptotic cells are not scavenged, they progress to a lytic and inflammatory phase called secondary necrosis. The mechanism by which this occurs is unknown. Here we show that caspase-3 cleaves the GSDMD-related protein DFNA5 after Asp270 to generate a necrotic DFNA5-N fragment that targets the plasma membrane to induce secondary necrosis/pyroptosis. Cells that express DFNA5 progress to secondary necrosis, when stimulated with apoptotic triggers such as etoposide or vesicular stomatitis virus infection, but disassemble into small apoptotic bodies when DFNA5 is deleted. Our findings identify DFNA5 as a central molecule that regulates apoptotic cell disassembly and progression to secondary necrosis, and provide a molecular mechanism for secondary necrosis. Because DFNA5-induced secondary necrosis and GSDMD-induced pyroptosis are dependent on caspase activation, we propose that they are forms of programmed necrosis.

## Abstract (web summary)

DFNA5 is related to the caspase-dependent pyroptosis inducer gasdermin D. Here the authors find that DFNA5 is cleaved by caspase 3 and show this cleavage skews cells away from apoptosis into secondary necrosis, a form of cell death characterized by membrane ballooning similar to pyroptosis.

Programmed cell death (PCD) pathways have important physiological roles in growth, survival, homeostasis and innate immunity of all multicellular organisms. Two important, yet phenotypically distinct, forms of PCD include apoptosis and programmed necrosis^1^. Although apoptosis is immunologically ‘silent', programmed necrosis is an inflammatory form of PCD characterized by cellular swelling, lysis and release of pro-inflammatory molecules^1^. Programmed necrosis is mediated by two distinct signalling pathways; the necroptotic pathway induces necroptosis and the pyroptotic pathway induces pyroptosis^1^. Necroptosis is triggered by activation of receptor-interacting protein kinase-3 (RIPK3), which phosphorylates the pseudokinase MLKL, causing it to translocate to the plasma membrane to induce cell permeabilization^2^. Pyroptosis is triggered primarily by activation of the inflammatory caspases, which include caspase-1 and caspase-11 (caspase-4/-5 in humans)^3^,^4^. Caspase-1 is activated by multiprotein complexes assembled by several proteins such as NLRP3, NLRC4, AIM2, pyrin and NLRP1, collectively referred to as canonical inflammasomes (reviewed in^5^). By contrast, human caspase-4 and -5, and their mouse ortholog caspase-11, are activated within non-canonical inflammasome complexes by directly binding to lipopolysaccharide from Gram-negative bacteria^4^. Studies have demonstrated that on activation of inflammatory caspases by the canonical and non-canonical pathways these caspases cleave a cellular substrate called gasdermin D (GSDMD) after Asp276 (refs 6, 7, 8), generating a necrotic N-terminal fragment capable of inducing pyroptosis by forming pores in the plasma membrane^9^,^10^,^11^,^12^.

In contrast to programmed necrosis, apoptosis is a non-inflammatory form of PCD mediated by activation of the apoptotic caspases and can occur either via an extrinsic or an intrinsic pathway^13^,^14^. Although the extrinsic pathway is activated by signalling through cell surface death receptors, the intrinsic pathway is activated by mitochondrial damage. However, both pathways converge on the activation of the executioner caspases (caspase-3, 6 and 7), which target >600 substrates to orchestrate morphological changes associated with apoptosis^14^. At the terminal stage of apoptosis, cells are phagocytosed *in vivo* by scavenger cells, such as macrophages or neutrophils. However, if these cells are not removed in a timely fashion, as is the case *in vitro*, they progress to a final phase called secondary necrosis characterized by cytoplasmic swelling and plasma membrane damage, similar to the phenotype of cells undergoing pyroptosis or necroptosis^15^,^16^. The mechanism of secondary necrosis and whether it is mediated by substrates of apoptotic caspases is not clear.

DFNA5 belongs to the same gasdermin superfamily, as GSDMD and has been implicated in the induction of cell death and as a putative tumour suppressor^17^,^18^,^19^. Mutations in intron 7 of the *DFNA5* gene have been shown to cause sensorineural hearing loss because of skipping of exon 8 at the pre-mRNA level and the translation of a C-terminally truncated protein^20^,^21^. Although the full-length product does not have cytotoxic activity, the truncated form does^18^,^19^. In addition, promoter hypermethylation leading to DFNA5 inactivation has been detected in 52% of primary gastric cancers making it a putative tumour suppressor and further suggesting a role in promoting cell death^22^. Finally, expression of DFNA5 is induced by the transcription factor p53 in response to etoposide, a potent inducer of apoptosis^23^.

Because DFNA5 is related to GSDMD, and its C-terminal truncation has been shown to cause cell death^18^,^19^, we investigated whether it is cleaved by apoptotic caspases to induce secondary necrosis. Here we show that DFNA5 is a physiological substrate for caspase-3. Mechanistically, caspase-3 cleaves DFNA5 after Asp270 to generate a necrotic DFNA5-N fragment that translocates to the plasma membrane to permeabilize it and induce secondary necrosis/pyroptosis. In 293T cells that stably express DFNA5, activation of caspase-3 by stimulation of the mitochondrial apoptotic pathway with Bax overexpression, or infection by the apoptosis-inducing vesicular stomatitis virus (VSV) or encephalomyocarditis virus (ECMV) results in cleavage of DFNA5 and induction of secondary necrosis. Similarly, in WT and caspase-1/caspase-11 (casp-1/11)-deficient macrophages, infection with VSV or treatment with etoposide results in cleavage of endogenous DFNA5 into the necrotic N-terminal fragment and induction of secondary necrosis. Deletion of DFNA5 in macrophages mostly inhibits VSV-induced and etoposide-induced secondary necrosis. Interestingly, unlike WT cells, DFNA5-deficient cells do not swell, but extensively disassemble into small apoptotic bodies. Combined, our results indicate that DFNA5 regulates disassembly and progression of apoptotic cells to secondary necrosis on cleavage by caspase-3.

## Results

### DFNA5 is specifically cleaved by caspase-3

Our studies in VSV-infected immortalized bone marrow-derived macrophages (BMDMs) from casp-1/11-deficient mice revealed that these macrophages undergo a caspase-dependent necrotic form of cell death resembling pyroptosis. VSV-infected casp-1/11-deficient macrophages released high amount of LDH in the culture supernatants and showed microscopic features of plasma membrane swelling characteristic of necrosis/pyroptosis (Supplementary Fig. 1). Pharmacological inhibition of the necroptotic pathway with the RIPK3 inhibitor GSK'872 alone did not block this cell death. However, combined treatment with GSK'872 and the pancaspase-inhibitor zVAD-fmk completely blocked cell death, indicating that this VSV-induced necrotic-like cell death is not mediated by necroptosis but is mediated by caspases other than caspase-1 and caspases-11. The observed high LDH release in the presence of zVAD-fmk is caused by zVAD-fmk-induced necroptosis, because inhibition of caspases leads to activation of the RIPK3-MLKL necroptotic cell death pathway in macrophages^24^. Since casp-1/11 dKO macrophages lack inflammatory caspases, which induce pyroptosis by cleaving GSDMD (refs 6, 7, 8), these results suggest that the observed VSV-induced necrosis in these cells is likely the result of non-inflammatory caspase activity on substrates other than GSDMD. To test this hypothesis we investigated whether non-inflammatory caspases can cleave other GSDMD-related family members to induce necrosis-like phenotype.

**Figure 1:**
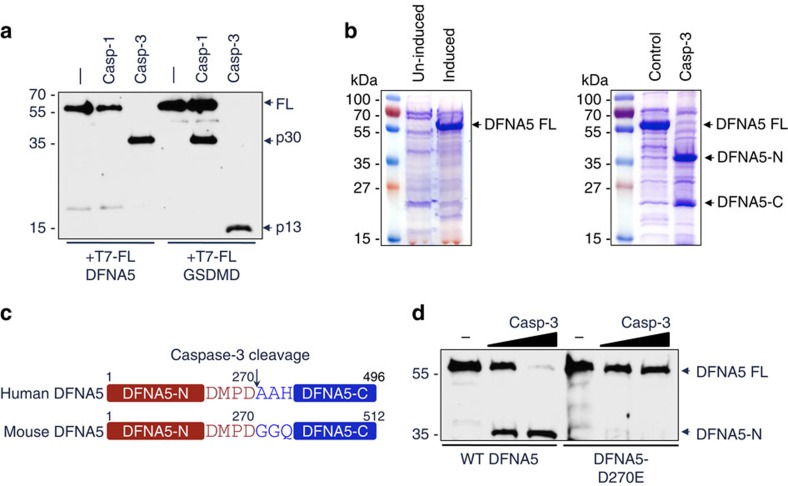
Caspase-3 cleaves DFNA5 after Asp270. (**a**) Immunoblot of purified N-terminal His6-T7-tagged DFNA5 and GSDMD proteins incubated without (−) or with caspase-1 (casp-1) or caspase-3 (casp-3) for 45 min at 37 °C. The blot was probed with anti-T7 antibody. (**b**) Coomassie stained SDS-polyacrylamide gels of TALON-immobilized proteins from uninduced or IPTG-induced BL-21 pET28b-DFNA5 bacteria (left panel), or IPTG-induced BL-21 pET28b-DFNA5 bacteria incubated without (control) or with caspase-3 for 45 min (right panel). The caspase-3-generated N- and C-terminal DFNA5 fragments (DFNA5-N and DFNA5-C, respectively) are indicated. (**c**) Diagrammatic representation of human and mouse DFNA5 proteins showing the caspase-3 recognition motif at aa 267–270. (**d**) Immunoblot of purified N-terminal T7-tagged WT DFNA5 and DFNA5-D270E proteins incubated without (−) or with increasing amounts of caspase-3 for 45 min. The blot was probed with anti-T7 antibody. Results are representative of at least three independent experiments.

DFNA5, a GSDMD-related family member, shares only ∼28% identity with GSDMD within the region corresponding to the pyroptotic GSDMD N-terminal domain (Supplementary Fig. 2). Nevertheless, genetic mutations within intron 7 of human DFNA5 that cause skipping of exon 8 and truncation of the C-terminus of DFNA5 at residue 315 leads to hearing loss^21^, suggesting that the N-terminal domain of DFNA5, like GSDMD N-terminal domain, may possess cell-death inducing activity. As a first step to investigate whether DFNA5 is an effector of necrotic-like cell death downstream of caspase activation, we tested whether DFNA5 is a target for caspases. Incubation of N-terminal T7 tagged recombinant DFNA5 with caspase-3, but not with caspase-1, resulted in the generation of ∼35 kDa N-terminal fragment similar in size to the caspase-1 generated GSDMD N-terminal domain (Fig. 1a). Among human caspases only caspase-3 was able to efficiently cleave DFNA5 (Supplementary Fig. 3a). Notably, caspase-3 was also capable of cleaving GSDMD but resulted in the generation of a 13 kDa GSDMD N-terminal fragment. This result is consistent with a previous report^25^, which showed that GSDMD is cleaved at Asp87 during apoptosis (http://wellslab.ucsf.edu/cgibin/retrieve_1.0.cgi?degrabase_type=both&p1_type=all&substrate=gsdmd). This residue is part of a consensus caspase-3 cleavage site (DxxD) and is conserved in both human and mouse GSDMD. The physiological consequence of this cleavage remains to be investigated. Cleavage of DFNA5 by caspase-3 was very efficient and complete, and appears to occur only at a single site because only two fragments (DFNA5-N and DFNA5-C) were generated when the cleaved products were visualized by Coomassie stain (Fig. 1b, right panel).

To identify the caspase-3 recognition site in DFNA5 we searched for a consensus caspase-3 recognition motif (DxxD) in the linker region between the DFNA5-N and DFNA5-C domains. We found that both human and mouse DFNA5 proteins contain putative caspase-3 recognition motifs at residues 267–270 (267DMPD270) (Fig. 1c). To confirm that this site is indeed cleaved by caspase-3, we substituted Asp270 with Glutamate in human DFNA5 by site-directed mutagenesis. The resulting D/E mutant DFNA5 was completely resistant to cleavage by caspase-3 (Fig. 1d). We further subjected the DFNA5-C fragment to Edman degradation and obtained the N-terminal sequence AAHGI, which exactly matches the N-terminal sequence of the predicted DFNA5-C fragment generated by cleavage of human DFNA5 after Asp270.

**Figure 2:**
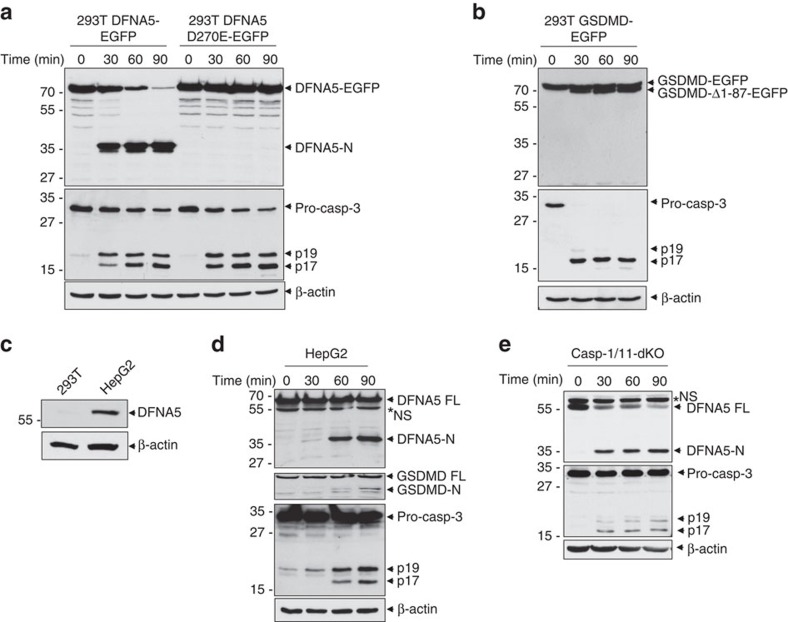
DFNA5 is cleaved by caspase-3 downstream of the Apaf-1 apoptosome. (**a**,**b**) Immunoblots of S100 lysates from stable 293T-DFNA5-EGFP and 293T-DFNA5-D270E-EGFP cells (**a**), or stable 293T-GSDMD-EGFP cells (**b**) stimulated with cytochrome c for the indicated times at 37 °C. The blots were probed with anti-DFNA5 (**a**, upper), anti-GSDMD (**b**, upper), anti-caspase-3 (**a**,**b**, middle) or anti-β-actin (**a**,**b**, lower) antibodies. (**c**) Immunoblot of endogenous DFNA5 in 293T and HEPG2 total cell lysates. (**c**,**d**) Immunoblots of S100 lysates from human HEPG2 (**d**) or mouse casp-1/casp-11-double knockout (casp-1/11-dKO) (**e**) macrophages stimulated with cytochrome c for the indicated times at 37 °C. The blots were probed with anti-DFNA5 (upper in **d**,**e**), anti-GSDMD (second from top in **d**), anti-caspase-3 (third from top in **d**, middle in **e**) or anti-β-actin (fourth from top in **d**, lower in **e**) antibodies. Asterisk indicate non-specific band (NS). Results are representative of at least three independent experiments.

To show that DFNA5 is a physiological target for caspase-3 after its activation by the Apaf-1 apoptosome, we stimulated S100 cell extracts from 293T cells stably expressing a C-terminal EGFP-tagged WT or D270E DFNA5 proteins (293T-DFNA5-EGFP cells or 293T-DFNA5-D270E-EGFP cells, respectively) with cytochrome c. Notably, activation of endogenous caspase-3 within the Apaf-1 apoptosome by cytochrome c resulted in robust processing of WT DFNA5 but not the D270E DFNA5 mutant (Fig. 2a). Caspase-3 activation by cytochrome c in S100 extracts from 293T cells stably expressing GSDMD-EGFP (293T-GSDMD-EGFP cells) also resulted in processing of GSDMD at Asp87 (Fig. 2b). No DFNA5 or GSDMD processing was observed in S100 lysates from the caspase-3-deficient MCF7 breast cancer cell line^26^, indicating that caspase-3 is the primary protease responsible for processing of DFNA5 and GSDMD downstream of the apoptosome (Supplementary Fig. 3b, left panels, and Supplementary Fig. 3c). Consistent with this, DFNA5 processing was restored in MCF-7 cells stably expressing caspase-3 (Supplementary Fig. 3b, right panels). Similarly, activation of S100 extracts from HEPG2 cells which unlike 293T cells express detectable endogenous DFNA5 protein (Fig. 2c), or from immortalized caspase-1/11-double knockout macrophages, resulted in processing of endogenous DFNA5 and GSDMD (Fig. 2d,e). Combined, these results indicate that caspase-3 specifically cleaves DFNA5 after Asp270 and that DFNA5 is a physiological target of caspase-3 downstream of the Apaf-1 apoptosome.

### The processed DFNA5-N fragment has a necrotic activity

**Figure 3:**
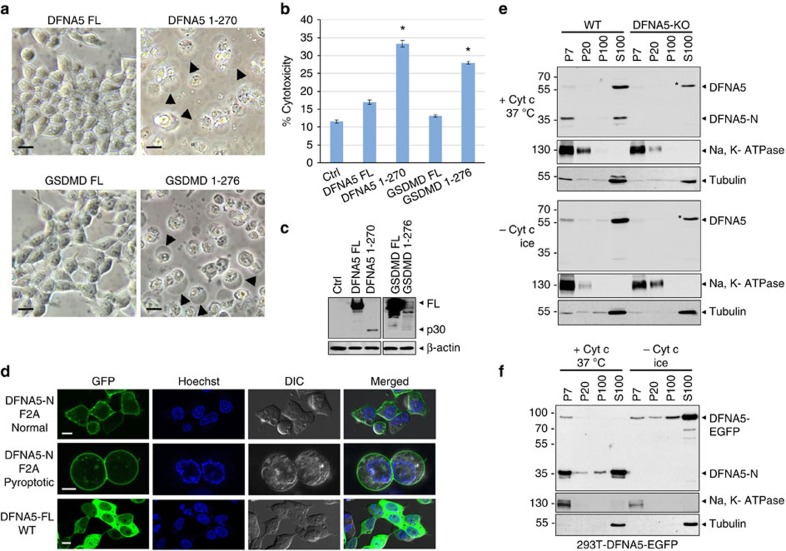
The cleaved N-terminal DFNA5 fragment has intrinsic necrotic activity. (**a**) Microscopic images of 293T cells transfected with expression constructs for full-length DFNA5 (DFNA5 FL), full-length GSDMD (GSDMD FL), DFNA5-N (DFNA5 1–270) or GSDMD-N (GSDMD 1–276) as indicated. Arrowheads indicate ballooned cell membrane characteristic of necrotic/pyroptotic cells. Scale bar, 20 μm. (**b**) Cytotoxicity of DFNA5 and GSDMD and their fragments as measured by LDH release in the culture supernatants of 293T cells transfected with the indicated expression constructs for DFNA5 and GSDMD. (*n*=3) *P<0.001, Student's *t*-test. (**c**) Immunoblots showing the expression of the proteins in (**b**). The lower expression of DFNA5 1–270 and GSDMD 1-276 fragments is due to their toxicity. (**d**) Confocal images of the DFNA5-N-F2A-EGFP mutant (upper and middle), or full-length WT DFNA5-EGFP expressed in 293T. The upper panels show normal cells and the middle panels show necrotic/pyroptotic cells. For videos of the cells shown in the middle panels see Supplementary Videos 1–3. Scale bar, 10 μm. (**e**,**f**) Immunoblots of cytochrome c-activated (+ Cyt c, 37° C) or unactivated (− Cyt c, ice) cell lysates from *DFNA5*^*+/+*^ (WT) and *DFNA5*^*−/−*^ (DFNA5-KO) macrophages (**e**) or 293T-DFNA5-EGFP cells (**f**) probed with anti-DFNA5 (upper), anti-Na, K-ATPase (middle) or anti-tubulin (lower) antibodies after fractionation into P7 (heavy membrane), P20 (light membrane), P100 (insoluble cytosol) and S100 (soluble cytosol). Asterisk indicates non-specific band with similar size/migration as full-length DFNA5. Results are representative of at least three independent experiments. Error bars represent standard error of the mean (s.e.m).

To investigate whether the caspase-3-generated DFNA5-N fragment has a necrotic activity like the GSDMD-N fragment that is generated by inflammatory caspases, full-length DFNA5 and GSDMD and their processed fragments DFNA5-N and GSDMD-N, respectively, were ectopically expressed in 293T cells. In contrast to full-length proteins, both DFNA5-N and GSDMD-N fragments induced extensive cell death with characteristic morphological and biochemical features of necrosis as evidenced by ballooning of the cell membrane and LDH release, respectively (Fig. 3a,b). Because of their potent killing activity the expression levels of DFNA5-N and GSDMD-N fragments were barely detectable compare to full-length proteins (Fig. 3c). Induction of necrosis by DFNA5-N and GSDMD-N fragments was largely not inhibited by the pan-caspase inhibitor zVAD-fmk, indicating that these proteins do not require caspase activity to induce cell death (Supplementary Fig. 4a). However, there was a slight reduction in LDH and HMGB1 release in the zVAD-fmk treated cells (Supplementary Fig. 4b,c), suggesting that caspase activation occurs during this form of necrosis and may contribute to enhancement of cell death.

To determine the minimal sequence of DFNA5-N fragment capable of inducing necrosis, we made progressive N-terminal and C-terminal deletion mutants of human DFNA5-N and mouse GSDMD-N. Expression of these deletion mutants in 293T cells revealed that the first 4N-terminal residues of DFNA5-N are critical for its killing activity (Supplementary Fig. 5a). Deletion of the homologous residues in GSDMD (residues 5–7) also inactivated GSDMD-N killing activity (Supplementary Fig. 5b). This region contains a phenylalanine residue (F2 in DFNA5; F4 in GSDMD), mutation of which to alanine dramatically increased the expression of this mutant but significantly reduced its killing activity (Supplementary Fig. 5a,c). Progressive deletions at the C-termini of DFNA5-N and GSDMD-N fragments revealed that residues 235–270 of DFNA5-N fragment or residues 232–276 of GSDMD-N fragment are not important for their killing activity. Interestingly, deletion of the last phenylalanine (F234) in the DFNA5–1–234 fragment or deletion of the last leucine (L231) in the GSDMD-1–231 fragment completely inactivated these proteins. Collectively these results indicate that the caspase-3 generated DFNA5-N fragment has an intrinsic necrotic activity, and that the N-terminal F2 and C-terminal F234 residues are critical for this activity.

### DFNA5-N targets the plasma membrane

The intrinsic ability of DFNA5-N and GSDMD-N to induce plasma membrane swelling and necrosis suggests that these proteins target the plasma membrane. To provide evidence that DFNA5-N targets the plasma membrane we generated a construct encoding a C-terminal-EGFP tagged DFNA5-N fragment. Ectopic expression of this protein in 293T cells induced rapid and extensive necrosis, which hampered clear assessment of its subcellular localization. However, ectopic expression of an EGFP-tagged DFNA5 F2A mutant which has reduced killing activity and increased expression (Supplementary Fig. 5a) allowed clear visualization of DFNA5-N on the plasma membrane (Fig. 3d). This DFNA5 mutant showed membrane localization with some cytoplasmic distribution in healthy cells (upper panels) but mainly membrane localization in necrotic cells (Fig. 3d middle panels and Supplementary Videos 1–3). The full-length DFNA5 protein showed exclusive cytoplasmic localization (Fig. 3d, lower panels).

To provide additional evidence that the DFNA5-N fragment targets the plasma membrane, we activated DFNA5 by incubation of cell lysates from WT macrophages or 293T-DFNA5-EGFP cells with cytochrome c and then fractionated the cell lysates into membrane and cytosolic fractions by centrifugation. Consistent with the confocal microscopy results, the activated endogenous DFNA5-N fragment from WT macrophages and the exogenous DFNA5-N fragment from 293T-DFNA5-EGFP were found in the heavy membrane and S100 fractions, whereas the unactivated full-length DFNA5 was found almost exclusively in the S100 fraction (Fig. 3e,f). These results are similar to those reported for GSDMD recently^11^. Altogether, these results strongly suggest that DFNA5-N fragment, like the GSDMD-N fragment, targets the plasma membrane to induce necrosis.

### DFNA5 N-terminus harbours a membrane targeting domain

While this work was under review several recent reports showed that N-terminal fragments of members of the gasdermin family indeed target the plasma and mitochondrial membranes through interactions with membrane lipids leading to formation of large pores^9^,^10^,^11^,^12^. Ding *et al*. also reported the crystal structure of GSDMA3 (ref. 10). We generated a homology model of DFNA5 (Supplementary Fig. 6a) using SWISS-MODEL^27^ based on the crystal structure of GSDMA3 (Supplementary Fig. 6b)^10^, which is 19.7% sequence identical to DFNA5. Both proteins are built by two arches closed like a clamp to bury an N-terminal moiety enriched in hydrophobic and basic residues (coloured in red in Supplementary Fig. 6a,b). Caspase-3 cleavage releases DFNA5 N-terminal arch (res. 1–270) (Supplementary Fig. 6c) that, like GSDMA3 (ref. 10), has no apparent structural similarity to known proteins in the RCSB database. However, removing the N-terminal residues 1–57 that pack loosely against DFNA5 concave surface, reveals a seahorse-shaped fold (Supplementary Fig. 6d) characterized by a four stranded, highly twisted β-sheet decorated with small clusters of α-helices, characteristic of the Membrane Attack Complex/Perforin-like Family (MACPF) (Supplementary Fig. 6e,f)^28^,^29^. Accordingly, a DALI search^30^ identified several members of the membrane attack complex/perforin-like family super-family such as astrotactin (Z-score=3.1), the complement component C8a protein (‘Poly-C8') (Z-score=2.4) and Pleurotolysin B (Z-score=2.0) as *bona fide* structural homologues of DFNA5-core. Interestingly, the structure of the oligomeric pore-forming Poly-C9 component of the complement membrane attack complex (MAC) was recently determined using cryo-electron microscopy single particle analysis, revealing a 22-fold symmetric ring of C9 molecules^31^ (Supplementary Fig. 7a–c). Poly-C9 heterodimerizes with the closely related Poly-C8 subunit and other subunits to form the MAC, a multi-protein complex that forms pores in the membrane of target pathogens^32^. The ability of these proteins to penetrate and insert into biomembranes is based on two helices (known as TMH1 and TMH2 and shown in red in Supplementary Fig. 7a–c), which change conformation to form anti-parallel β-hairpin, generating an 88-strand pore-forming β-barrel (shown in red in Supplementary Fig. 7c for only one protomer). Using the structural similarity between DFNA5 and Poly-C8/C9, we generated a model of the putative pore-conformation of DFNA5 (Supplementary Fig. 7d,e), which is ∼125 Å in diameter. In analogy to other pore-forming toxins^33^, we propose DFNA-5N-terminal moiety undergoes a complex conformational change upon oligomerization that results in formation of a membrane penetrating-tip (Supplementary Fig. 7f).

**Figure 4:**
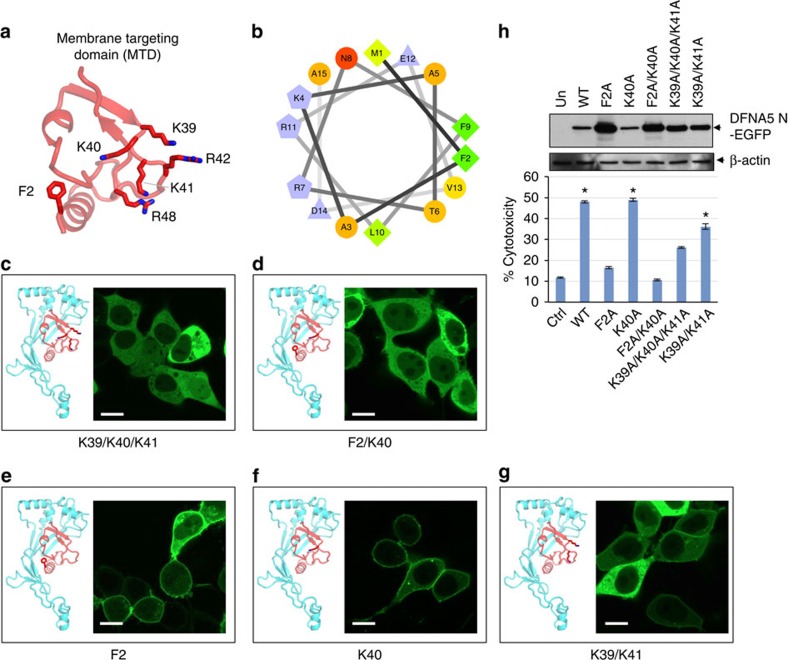
Molecular determinants of plasma membrane targeting of DFNA5-N. (**a**) Predicted structure of DFNA5 MTD spanning residues 1–57 showing the side chains of residues F2, K39, K40, K41, R42 and R48 as sticks. (**b**) Helical wheel representations of DNFA5 residues 1–14 showing the putative amphipathic nature of the N-terminal helix. K4, R11 and R7 are clustered on the left while F2 and F9 are on the right hand side of the helix. (**c**–**g**) Confocal images of the indicated DFNA5-N-EGFP alanine mutants expressed in 293T. The ribbon diagrams on the left of each image show the position of side chains of residues mutated to alanines. Scale bar, 10 μm. (**h**) Cytotoxicity of the indicated DFNA5-N mutants as measured by LDH release in the culture supernatants of 293T cells transfected with constructs encoding these mutants (*n*=3). *P<0.001, Student's *t*-test. Expression of these mutants in cell lysates is shown in the top panel as measured by immunoblot analysis with anti-DFNA5 antibody. Results are representative of at least three independent experiments. Error bars represent standard error of the mean (s.e.m).

Residues 1–56 of DFNA-5 (Fig. 4) contain an amphipathic α-helix and a β-hairpin exposing a basic patch (39-KKKR-42); overall this domain contains 19 hydrophobic residues that we propose are involved in membrane targeting and penetration (we refer to this region of DFNA5 as Membrane Targeting Domain or MTD) (Fig. 4a,b). In analogy to the Poly-C9 component of the MAC complex^31^, we speculate that DNFA5 MTD also undergoes conformational changes upon insertion into the plasma-membrane (Supplementary Fig. 7f). Supporting this model, point mutations of K39, K40 and K41 to alanines almost completely prevented targeting of DFNA5-N to the plasma membrane and significantly reduced its necrotic activity (Fig. 4c,h). Combined mutation of K39 and K41 to alanines also largely prevented targeting of this mutant to the plasma membrane and significantly reduced its necrotic activity (Fig. 4g,h). However, mutation of K40 to alanine did not significantly reduce its necrotic activity but showed both membrane and cytoplasmic localization similar to the F2A mutant (Fig. 4f,h). Interestingly, a combined mutation of F2 and K40 to alanines resulted in total cytoplasmic localization and no necrotic activity (Fig. 4d,h). The significantly reduced necrotic activity and increased expression of the F2A mutant compared with WT or K40A mutant suggest that this residue might also be critical for DFNA5-N oligomerization. These results indicate that residues 1–56 of DFNA5 contain the MTD of DFNA5 and may also facilitate its oligomerization. Unlike known pore-forming toxins that assemble pores from the outside of the plasma-membrane, DFNA5 is a cytoplasmic protein that must assemble oligomers and penetrate the plasma-membrane from the inside. This is likely dictated by the lipid composition of the inner membrane. The molecular details of DFNA5 cell-penetration remain to be investigated in future studies.

### DFNA5 is activated by the mitochondrial apoptotic pathway

**Figure 5:**
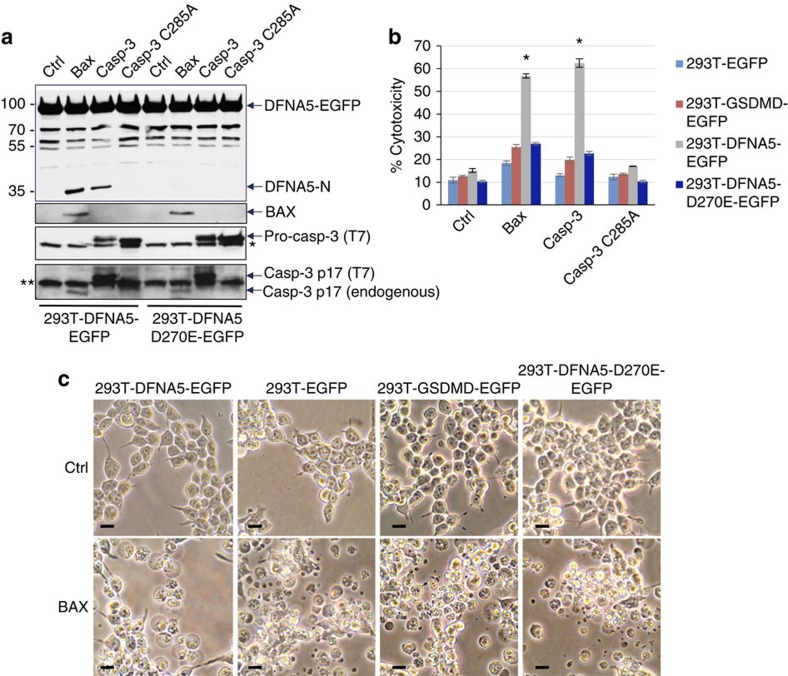
Activation of DFNA5 downstream of the mitochondrial apoptotic pathway. (**a**) Immunoblots of cell lysates from stable 293T-DFNA5-EGFP and 293T-DFNA5-D270E-EGFP cells transfected with vector control (Ctrl) or constructs for Bax, constitutively active T7-tagged caspase-3 (casp-3) or inactive caspase-3 (casp-3-C285A) for 24 h as indicated. The blots were probed with anti-DFNA5 (upper), anti-Bax (middle), or anti-caspase-3 (lower two panels) antibodies. Asterisk indicates endogenous procaspase-3. Double asterisks indicate non-specific band. (**b**) Cytotoxicity of Bax and constitutively active caspase-3 as measured by LDH release in the culture supernatants of the indicated 293T cell lines. (*n*=3) *P<0.0001), Student's *t*-test. (**c**) Microscopic images of the indicated 293T cell lines transfected with a vector control (Ctrl) or an expression construct for full-length Bax (BAX). Scale bar, 20 μm. Results are representative of at least three independent experiments. Error bars represent standard error of the mean (s.e.m).

Intriguingly, a process called secondary necrosis has been observed following activation of the apoptotic programme in many cell types^16^,^34^. Secondary necrosis refers to a terminal phase at the end of the apoptotic programme characterized by plasma membrane permeabilization, swelling and lysis, three features that are also common to pyroptosis and necroptosis^16^,^34^,^35^,^36^. This process occurs if the full apoptotic programme is completed and there is no intervention of scavengers such as seen under *in vitro* conditions, or when there is too much apoptosis *in vivo* that overwhelms the available scavenging capacity. To demonstrate a role for cleaved DFNA5 in secondary necrosis downstream of the mitochondrial apoptotic pathway we stably reconstituted 293T cells, which do not express detectable endogenous DFNA5 protein (Fig. 2c), with DFNA5-EGFP or DFNA5-D270E-EGFP. We then transfected these cells with an expression construct for Bax, a proapoptotic Bcl-2 family member that activates the mitochondrial apoptotic pathway by inducing cytochrome c release from the mitochondria. As shown in Fig. 5a, expression of Bax in the WT DFNA5-EGFP-reconstituted 293T cells led to processing of the DFNA5-EGFP protein to generate the active DFNA5-N fragment. However, expression of Bax in the DFNA5-D270E-EGFP-reconstituted 293T cells did not induce processing of this protein (Fig. 5a). Consistent with a role for DFNA5-N fragment in inducing necrosis, Bax expression also caused significant LDH release from the 293T-DFNA5-EGFP cells but not from the cells expressing the D270E mutant DFNA5-EGFP or GSDMD-EGFP proteins (293T-GSDMD-EGFP cells) (Fig. 5b). The 293T-DFNA5-EGFP cells also showed microscopic features of necrosis (ballooning of the cell membrane), which were largely absent in the cells expressing the D270E mutant DFNA5-EGFP, GSDMD-EGFP or EGFP proteins (Fig. 5c).

Consistent with the above results transfection of 293T-DFNA5-EGFP cells with a constitutively active^37^, but not inactive mutant caspase-3 also led to processing of DFNA5 and significant induction of LDH release (Fig. 5a,b). No processing of DFNA5 or significant LDH release was observed in 293T-DFNA5-D270E-EGFP cells. In addition, no significant LDH release was observed in the GSDMD-EGFP-expressing 293T-GSDMD-EGFP cells after transfection with active caspase-3 (Fig. 5b). Combined these results show that DFNA5, but not GSDMD, mediates secondary necrosis downstream of the mitochondrial apoptotic pathway and caspase-3 activation.

### DFNA5 is activated by viral infection and etoposide

**Figure 6:**
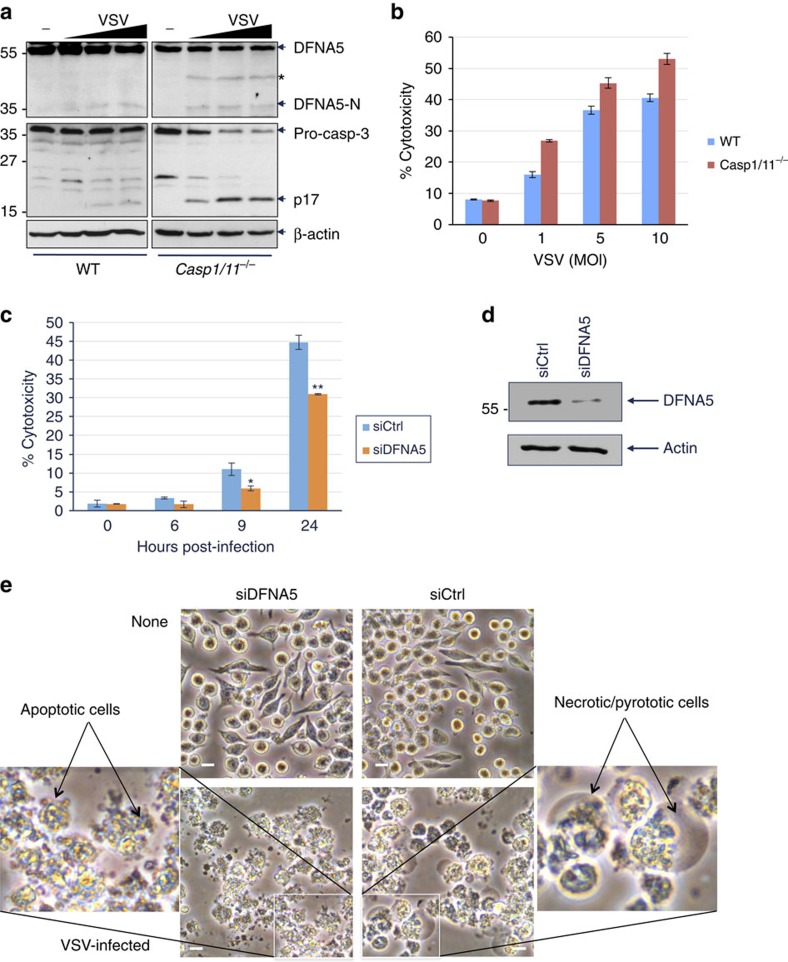
Activation of DFNA5 downstream of viral infection in macrophages. (**a**) Immunoblots of DFNA5 in cell lysates from uninfected (−) or VSV-infected (1, 5, 10 MOI) WT (left) or casp-1/11 dKO (right) immortalized BMDMs. The lower panels show caspase-3 and β-actin in the same cell lysates. Asterisk indicates non-specific band. (**b**,**c**) Cytotoxicity of VSV as measured by LDH release in the culture supernatants of WT and casp-1/11 dKO BMDMs infected with VSV for 12 h (**b**) (*n*=3) or casp-1/11 dKO BMDMs transfected with control siRNA (siCtrl) or DFNA5 siRNA (siDFNA5) for 48 h and then infected with VSV (1 MOI) for the indicated times (**c**). (*n*=3) *P<0.01, ^**^P<0.001, Student's *t*-test. (**d**) Immunoblots of DFNA5 in the control siRNA (siCtrl)- or DFNA5 siRNA (siDFNA5)-transfected casp-1/11 dKO BMDMs shown in (**c**). (**e**) Microscopic images of the control siRNA (siCtrl)- or DFNA5 siRNA (siDFNA5)-transfected casp-1/11 dKO BMDMs shown in (**c**). Scale bar, 20 μm. The insets show higher magnification images of apoptotic and necrotic cells. Results are representative of at least three independent experiments. Error bars represent standard error of the mean (s.e.m).

Infection with ssRNA viruses such as VSV or ECMV can lead to caspase-3 activation and cell death^38^,^39^. To investigate whether activation of caspase-3 by viral infection can lead to DFNA5-dependent secondary necrosis, WT and casp-1/casp-11-double knockout (casp-1/11 dKO) macrophages were infected with VSV and then subjected to western blot and LDH release assays to detect cleavage of DFNA5 and measure necrosis, respectively. As shown in Fig. 6a, VSV infection caused a dose-dependent processing of DFNA5 into the active DFNA5-N fragment in both WT and casp-1/11-dKO macrophages. Altogether with DFNA5 processing there was LDH release from both WT and casp-1/11-dKO macrophages (Fig. 6b). LDH release was slightly higher in the casp-1/11-dKO cells probably because of increased caspase-3 activation in these cells (Fig. 6a, lower panels). This is consistent with recent results showing that caspase-1 deficiency increases the activity of caspase-3 (ref. 8). These results show that VSV infection is associated with caspase-3 activation, DFNA5 cleavage and secondary necrosis.

To provide evidence that virus-induced necrosis is dependent on caspase-3-induced processing of DFNA5 and independent of inflammatory caspase activation, we knocked down DFNA5 with siRNA in casp-1/11-dKO macrophages and then infected them with VSV. VSV-induced LDH release and necrosis was significantly reduced when DFNA5 was knocked down in these macrophages (Fig. 6c–e). Interestingly, VSV infection induced extensive blebbing and apoptotic bodies and very little plasma membrane swelling (ballooning) in the DFNA5-knocked down cells compared with control cells which showed both ballooning and blebbing (Fig. 6e).

**Figure 7:**
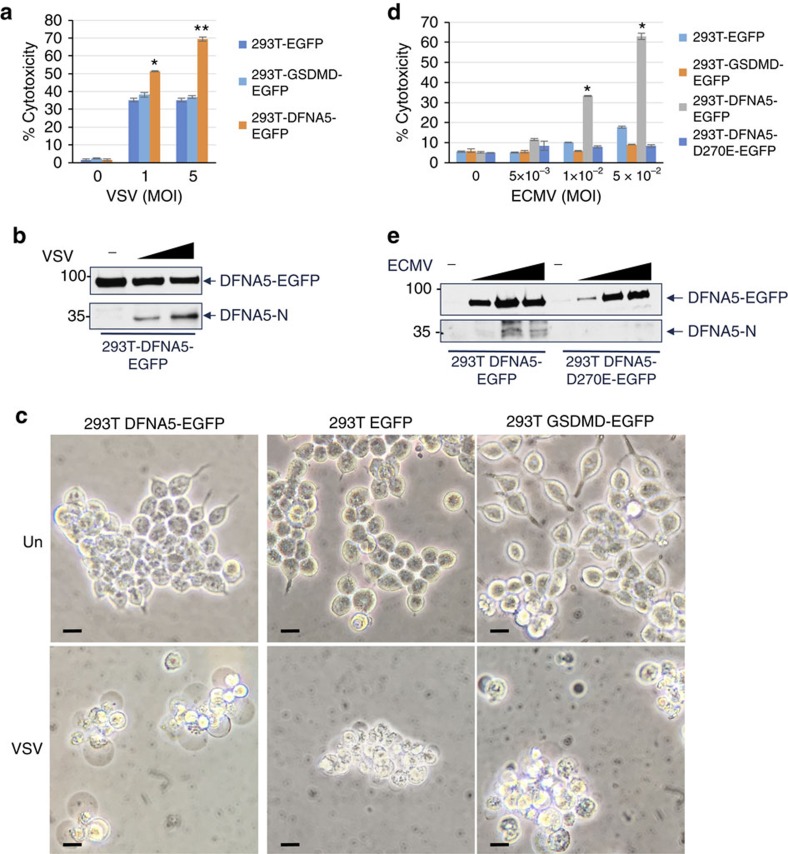
DFNA5 induces secondary necrosis downstream of viral infection in 293T cells. (**a**,**d**) Cytotoxicity of VSV (**a**) (*n*=3) (*P<0.001; ^**^P<0.0001, Student's *t*-test) or ECMV (**d**) (*n*=3) (*P<0.0001, Student's *t*-test) as measured by LDH release in the culture supernatants of the indicated 293T cell lines after 18 h infection with these viruses. (**b**,**e**) Immunoblots of DFNA5 in cell lysates from the indicated uninfected (−), VSV-infected (MOI: 1, 5 for 18 h) (**b**) or ECMV-infected (MOI: 0.005, 0.01, 0.05 for 18 h) (**e**) 293T cell lines. (**c**) Microscopic images of the indicated uninfected (Un) or VSV-infected (VSV) 293T cell lines. Scale bar, 20 μm. Results are representative of at least three independent experiments. Error bars represent standard error of the mean (s.e.m).

Similar to the results obtained with mouse macrophages, VSV infection lead to significantly more LDH release from 293T-DFNA5-EGFP cells compared with 293T or 293T-GSDMD-EGFP cells (Fig. 7a). DFNA5 was also cleaved in the VSV-infected 293T-DFNA5-EGFP and the cells exhibited the typical ballooning morphology associated with necrosis (Fig. 7b,c). Similar results were obtained when cells were infected with ECMV (Fig. 7d,e).

### DFNA5 is required for induction of secondary necrosis

**Figure 8:**
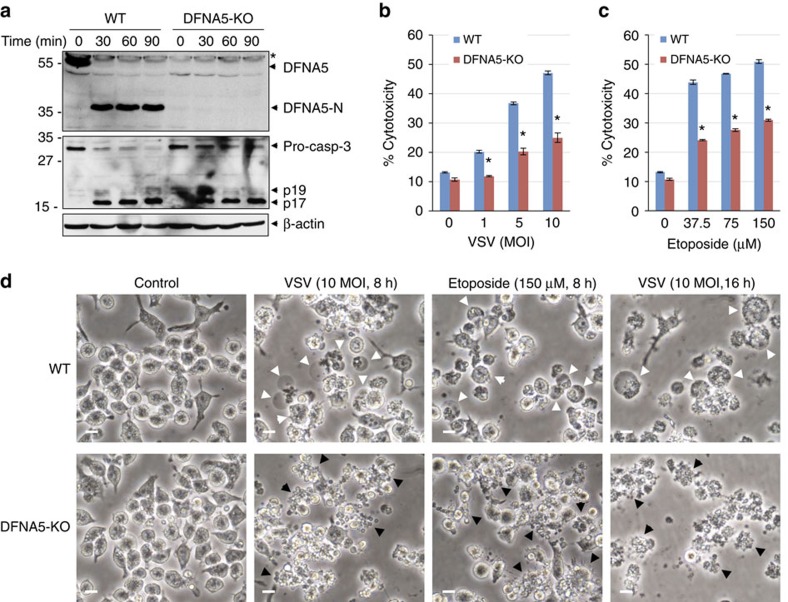
Knockout of DFNA5 reduces secondary necrosis in macrophages. (**a**) Immunoblots of S100 lysates from *DFNA5*^*+/+*^ (WT) and *DFNA5*^*−/−*^ (DFNA5-KO) macrophages stimulated with cytochrome c for the indicated times at 37 °C. The blots were probed with anti-DFNA5 (upper), anti-caspase-3 (middle) or anti-β-actin (lower) antibodies. Asterisk indicate non-specific band (NS). (**b**,**c**) Cytotoxicity of VSV (**b**) (*n*=3) and etoposide (**c**) (*n*=3) as measured by LDH release in the culture supernatants of *DFNA5*^*+/+*^ (WT) and *DFNA5*^*−/−*^ (DFNA5-KO) macrophages infected with VSV or treated with etoposide for 8 h. *P<0.0001, Student's *t*-test. (**d**) Microscopic images of *DFNA5*^*+/+*^ (WT) and *DFNA5*^*−/−*^ (DFNA5-KO) macrophages infected with VSV or treated with etoposide as indicated. White arrowheads indicate ballooning necrotic cells, and black arrowheads indicate blebbing apoptotic cells. Scale bar, 20 μm. Results are representative of at least three independent experiments. Error bars represent standard error of the mean (s.e.m).

To provide further evidence for the role of DFNA5 in induction of secondary necrosis in response to apoptotic triggers, we generated BMDM from *DFNA5*^*+/+*^ and *DFNA5*^*−/−*^ mice (Supplementary Fig. 8). Stimulation of S100 cell extracts from *DFNA5*^*+/+*^ macrophages with cytochrome c showed robust processing of DFNA5 into the DFNA5-N fragment which was completely absent in the *DFNA5*^*−/−*^ macrophages stimulated with cytopchrome c under the same conditions (Fig. 8a), confirming that *DFNA5*^*−/−*^ macrophages lack DFNA5. Consistent with the involvement of DFNA5 in VSV-induced necrosis, *DFNA5*^*−/−*^ macrophages showed significantly reduced LDH release and secondary necrosis compared with *DFNA5*^*+/+*^ or casp-1/11-DKO macrophages in response to VSV infection (Fig. 8b,d, Supplementary Fig. 9a). Similar reduction in LDH release and secondary necrosis was observed in *DFNA5*^*−/−*^ macrophages stimulated with the apoptotic agent etoposide (Fig. 8c,d). Processing of DFNA5 was also seen in *DFNA5*^*+/+*^ but not in *DFNA5*^*−/−*^ macrophages after VSV or etoposide treatments (Supplementary Fig. 9b,c). Similar to the results seen in casp-1/casp11-dKO macrophages (Fig. 6e), VSV infection or treatment with etoposide resulted in extensive blebbing and apoptotic bodies and very little plasma membrane swelling (ballooning) in the *DFNA5*^*−/−*^ macrophages compared with *DFNA5*^*+/+*^ control cells which showed both blebbing and ballooning (Fig. 8d; Supplementary Fig. 10a). This was clearly evidenced by time-lapse confocal microscopy, which showed that both etoposide treated *DFNA5*^*+/+*^ and *DFNA5*^*−/−*^ macrophages initiate the apoptotic programme with plasma membrane blebbing that progresses to plasma membrane swelling and ballooning (secondary necrosis) in *DFNA5*^*+/+*^ but not in *DFNA5*^*−/−*^ macrophages (Supplementary Videos 4 and 5). Since necrotic cells are permeable to the propidium iodide (PI) stain, flow cytometric analyses showed significantly more PI-positive and PI/Annexin V-double positive cells in etoposide-treated *DFNA5*^*+/+*^ macrophages than in etoposide-treated *DFNA5*^*−/−*^ macrophages (Supplementary Fig. 11), providing additional support for the role of DFNA5 in induction of secondary necrosis in response to apoptotic triggers. An additional feature of secondary necrosis is the release of active caspase-3 p17 fragment in the culture supernatant^40^,^41^. Consistent with this, apoptotic *DFNA5*^*+/+*^ macrophages but not *DFNA5*^*−/−*^ macrophages released caspase-3 p17 in the culture supernatant (supplementary Fig. 10b). Altogether these results indicate that DFNA5 mediates secondary necrosis downstream of caspase-3 activation in response to viral and apoptotic agents.

## Discussion

**Figure 9:**
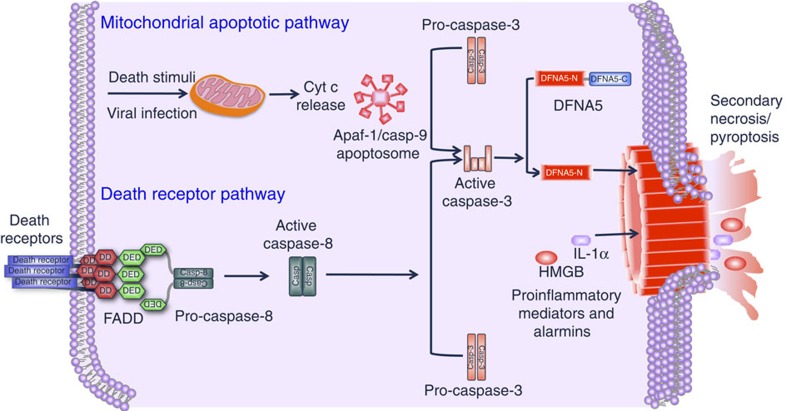
Signalling pathways leading to activation of DFNA5. Various death stimuli or viral infection can lead to permeabilization of the outer mitochondrial membrane causing the release of cytochrome c, which binds to Apaf-1 leading to assembly of the Apaf-1 apoptosome and activation of caspase-9. Within this complex active caspase-9 cleaves procaspase-3 to generate the active caspase-3 heterodimer. Active caspase-3 in turns cleaves DFNA5 at Asp270 to generate the necrotic DFNA5-N fragment which permeabilizes the plasma membrane by forming large pores causing osmotic lysis of the cell and releasing cellular contents including pro-inflammatory mediators and alamins, into the extracellular space. Caspase-3 can also be activated by the death receptor pathway, which is activated by death receptor ligands at the cell membrane. This pathway can potentially lead to caspase-3-mediated processing of DFNA5 and secondary necrosis.

Secondary necrosis has long been recognized as a terminal event following the completion of the apoptotic programme^34^. This occurs *in vitro* under tissue culture conditions or *in vivo* when apoptotic cells are not removed by scavenging cells such as in lysosomal disorders that impair removal of apoptotic cells by scavenging cells^42^ leading to secondary necrosis and inflammation. The main features of secondary necrosis are osmotic cell swelling and lysis, features that are also common to pyroptosis and necroptosis^16^,^34^,^35^,^36^. Like pyroptosis and necroptosis, secondary necrosis leads to leakage of the cell contents thereby it may cause tissue injury and induction of inflammation and other immune responses if the dying cells are not quickly removed by phagocytes. Secondary necrosis has been discounted as a non-specific and non-programmed osmotic swelling, and thus the molecular mechanism that leads to its onset remained poorly understood. Here we present compelling evidence that secondary necrosis is a form of programmed necrosis similar to pyroptosis, mediated by the GSDMD-related protein DFNA5. Our results clearly demonstrate that secondary necrosis is orchestrated by the activity of apoptotic caspase-3 which directly cleaves DFNA5 to produce a necrotic DFNA5-N fragment that targets and permeabilizes the plasma membrane. This new caspase-3- and DFNA5-dependent necrotic pathway is activated downstream of the mitochondrial apoptotic pathway and can potentially be activated downstream of the apoptotic death receptor pathway (Fig. 9). Considering the structural homology between DFNA5 and GSDMD and their mechanism of activation by caspases, our results suggest that the necrotic DFNA5-N fragment has an intrinsic pore-forming activity, similar to that of GSDMD-N fragment^9^,^10^,^11^,^12^, that damages and permeabilizes the plasma membrane.

Physiologically, formation of necrotic plasma membrane pores during secondary necrosis and pyroptosis may serve as a way to release a host of potent intracellular DAMPs (for example, HMGB1, ATP, inflammatory cytokines) that can act as alarm signals to activate and recruit immune cells to the site of apoptosis or infection. A recent study demonstrated that formation of the necrotic GSDMD pores also facilitate the trapping of intracellular pathogens within the largely intact plasma membrane, in a structure termed pore-induced intracellular trap (PIT)^43^. The PIT promotes efferocytosis of the cell corpse and entrapped pathogens by phagocytes^43^. These observations suggest that the GSDMD and likely the DFNA5 pores play important roles in the host innate immunity not only by releasing of alarmins but also by trapping pathogens within the cellular debris to be subsequently cleared by phagocytes. Our results show that VSV infection leads to activation of DFNA5 and induction of secondary necrosis, raising the possibility that the DFNA5 pores might also be involved in trapping of apoptotic pathogens like VSV, to allow for efficient efferocytosis of VSV-infected cells. Since both activated GSDMD and DFNA5 proteins have similar pore-forming activities, this suggests that they may have similar functions downstream of inflammatory and apoptotic caspases, respectively. This redundancy might be important for multicellular organisms considering that inflammatory caspases, GSDMD and DFNA5 might be differentially expressed in different cell types and tissues. It is also likely that DFNA5-N and GSDMD-N may function to target and kill different invading microorganisms as demonstrated recently for GSDMD-N (ref. 11). It is interesting that GSDMD-N fragment has a basic isoelectric point (pI=8.9) whereas DFNA5-N fragment has an acidic pI of 5.6 raising the possibility that they might have different specificities for different lipids and thus might kill different microorganisms. Future studies should clarify these points.

Although casp-1/11 dKO macrophages cannot cleave and activate GSDMD, they can still progress to a caspase-dependent secondary necrosis phenotype when infected with VSV. This progression is also dependent on DFNA5 as knockdown of DFNA5 in these macrophages attenuates their necrotic response to VSV-infection. This indicates that activation of the apoptotic caspase-3 in the absence of inflammatory caspases could serve to induce secondary necrosis through cleavage of DFNA5. Therefore, in addition to it being involved in secondary necrosis, the DFNA5 pathway might also serve as a backup pathway in the events of inflammasome inhibition by pathogen-mediated inflammasome-suppression strategies^44^,^45^. For example, cowpox virus and orthopoxviruses encode serpins such as CrmA that inhibit the activity of caspase-1, whereas myxoma virus and Shope fibroma virus encode pyrin-only proteins that inhibit inflammasome assembly^46^,^47^,^48^,^49^. Similarly, pathogenic bacteria produce virulence factors such as Yop proteins of Yersinia species to suppress the activity and activation of the inflammasome^50^.

Unlike secondary necrosis, apoptosis is a non-inflammatory and an immunologically silent form of cell death. This is likely because under *in vivo* conditions apoptotic cells are removed by scavenging cells before they fully activate DFNA5 and progress to secondary necrosis. Indeed, our results clearly demonstrate that there is a major difference in the outcome of the apoptotic programme between *DFNA5*^*+/+*^ and *DFNA5*^*−/−*^ macrophages. *DFNA5*^*+/+*^ macrophages initiate the apoptotic programm with membrane blebbing and terminate it with secondary necrosis, whereas *DFNA5*^*−/−*^ macrophages show only membrane blebbing and disassemble into small apoptotic bodies without plasma membrane swelling or LDH release and do not progress further (Supplementary Videos 4 and 5), even after 8 h treatment (Fig. 8d). Similarly, induction of apoptosis by VSV infection or activation of the mitochondrial apoptotic pathway by Bax in the largely DFNA5-deficient 293T cells only results in classic signs of apoptosis such as membrane blebbing, but when DFNA5 is stably expressed in these cells they progress to secondary necrosis. These findings indicate that DFNA5 regulates progression of apoptotic cells to secondary necrosis and may also function to prevent cellular disassembly into small apoptotic bodies during apoptosis. Since progression to secondary necrosis is linked to inflammatory diseases associated with defective clearance of apoptotic cells *in vivo*, this also raises the possibility that activation of DFNA5 might be a major contributing factor in these diseases.

DFNA5 has been implicated in hearing loss but the underling molecular mechanism involved in this process has not been elucidated. All DFNA5 mutations leading to hearing loss in humans have been attributed to exon 8 skipping at the pre-mRNA level leading to the translation of a C-terminally truncated DFNA5 protein^21^,^51^. Because ectopic expression of this mutant protein cause cell death, it has been proposed that the DFNA5 mutations represent gain of function mutations that increase the apoptotic activity of DFNA5 (ref. 51). Indeed, this conclusion is supported by our results which show that truncation of the C-terminus of DFNA5 by caspase-3 activates the necrotic activity of DFNA5. Thus, truncation and inactivation of the inhibitory DFNA5-C domain by hearing loss mutations unleash the necrotic activity of DFNA5 by mimicking processing of DFNA5 by caspase-3. This indicates that DFNA5 mutant-induced hearing loss is an outcome of disregulated secondary necrosis of hearing cells, raising the possibility that other forms of non-syndromic hearing loss might also be an outcome of disregulated secondary necrosis.

In addition to its involvement in hearing loss, DFNA5 has also been implicated in cancer as a tumour suppressor protein. Several studies showed that DFNA5 is silenced in many types of cancers by epigenetic mechanisms such as promoter methylation^22^,^52^,^53^. Reduced DFNA5 expression was shown to increase resistance of melanoma cells to etoposide-induced cell death, whereas increased DFNA5 expression by stable expression of DFNA5 in resistant cells resulted in increased etoposide-induced cell death^23^,^54^. DFNA5 is also a transcriptional target for p53 and its increased expression by p53 activation sensitizes tumour cells to cell death^23^,^55^. Our results indicate that DFNA5 functions directly downstream of caspase-3 activation to induce necrotic cell death. Therefore, the ability of caspase-3 to unleash the necrotic activity of DFNA5 by cleaving-off its inhibitory C-terminal DFNA5-C domain provides a mechanistic understanding of the tumour suppressive activity of DFNA5. This also places DFNA5 among the effector molecules that mediate the cell death activity of caspase-3. Since GSDMD is only present in birds and mammals, whereas DFNA5 is found in diverse species from teleost fishes to human^17^, the DFNA5 necrotic pathway might provide the same function in fish, reptile and amphibian species as the GSDMD pyroptotic pathway in birds and mammals. It is likely that the GSDMD pyroptotic pathway evolved recently perhaps to provide protection against new avian and mammalian pathogens.

In conclusion, we have identified DFNA5 as a key mediator of secondary necrosis and cellular disassembly downstream of apoptotic caspase-3. Future studies should elucidate whether activation of this pathway might serve as an innate immune pathway to induce secondary necrosis in response to infection with apoptotic pathogens and other apoptotic triggers, and whether it is responsible for inflammation associated with defective apoptotic cell clearance.

## Methods

### Antibodies and reagents

Rabbit polyclonal antibodies against caspase-1, NLRP3 and ASC were made in house and were described previously^56^,^57^,^58^. T7̇Tag monoclonal antibody HRP conjugate (Catalogue No. 69048) was from Novagen. Monoclonal anti-β-actin (Catalogue No. A-5316) was from Sigma Aldrich. Anti-DFNA5 (Catalogue No. sc-79233), anti-caspase-3 (Catalogue No. sc-7148), anti-GSDMD (Catalogue No. sc-393656) and anti-Bax (Catalogue No. sc-930) were from Santa Cruz. Anti- Na,K-ATPase polyclonal antibody (Catalogue No. 3010) was from Cell Signaling Technology. zVAD-fmk (Catalogue No. A1902) was obtained from ApexBio. GSK'872 (Cat No. AOB4887) was obtained from Aobious. CytoTox96 LDH-release kit (Cat No. G1780) was from Promega. TALON metal affinity resin (Catalogue No. 635502) and In-Fusion HD Cloning Plus (Catalogue No. 638910) were obtained from Takara Clontech. All antibodies were used at 1/1,000–1/2,000 dilutions for western blot analyses.

### Cell culture and treatments

Bone marrow-derived cells were harvested from the femurs of WT (C57BL/6) and knockout mice and differentiated into BMDMs by culturing in DMEM (GIBCO) medium supplemented with 10% FBS, 10 mM HEPES pH 7.0 (Invitrogen), 100 U ml^−1^ penicillin and streptomycin (complete DMEM) and 20% L929 supernatants in 10 cm dishes at 37 °C with 5% CO_2_ for 5–6 days. Immortalized BMDMs were generated by transformation of primary BMDMs with J2-CRE retrovirus (ref 24, 24). HepG2 cells were cultured in RMPI1640 medium (GIBCO) supplemented with 10% FBS, 10 mM HEPES pH 7.0 (Invitrogen), 100 U ml^−1^ penicillin and streptomycin, 1 mM sodium pyruvate (Cellgro), 2% sodium bicarbonate (Cellgro) and 0.1% beta-mercaptoethanol (GIBCO). MCF7 cells were cultured in complete DMEM, and 293T cells were cultured in DMEM F12 (GIBCO) medium supplemented with 10% FBS, 10 mM HEPES pH 7.0 (Invitrogen), 100 U ml^−1^ penicillin and streptomycin.

For viral infections, 293T cells or BMDMs were seeded in six-well plates overnight at a density of 4 × 10^5^ cells or 1 × 10^6^ cells per well, respectively. The cells were treated with VSV-GFP (Indiana strain) or ECMV in OPTI-MEMI medium. The culture supernatants and cells were collected at indicated times for analysis. In some experiments BMDMs were treated with zVAD-fmk (30 μM) or GSK'872 (10 μM) 3 h after infection.

For transfection experiments, 293T cells were seeded overnight in six-well plates at a density of 1 × 10^4^ cells per well. The next day, the culture medium was removed and replaced with 1 ml of OPTI-MEMI medium per well. Cells were transfected with 750 ng of plasmid DNA using Lipofectamine 2,000 (7 μl ml^−1^) as per the manufacturer's protocol (Invitrogen). The culture supernatants and cells were collected 24 h later for analysis.

### Generation of constructs and stable cell lines

All DFNA5 and GSDMD constructs were made in pcDNA3, pEGFPN1, pET28 vectors using In-Fusion PCR cloning kits (Takara Clontech) with appropriate PCR primers and full-length human DFNA5 or mouse GSDMD cDNAs. The human DFNA5 cDNA was obtained from TransOMIC (MGC premier cDNA clone for DFNA5—BC125065. Catalogue No. TCH1003). The mouse GSDMD cDNA was obtained from Dharmacon (MGC Mouse Gsdmd cDNA-Clone ID 4194837. Catalogue No. MMM1013–202765078). BL21 bacteria expressing His6-T7-DFNA5 or His6-T7-GSDMD under an IPTG inducible promoter were made by transforming BL21 bacteria with pET28b-DFNA5 or pET28b-GSDMD plasmids, respectively. Stable 293T-EGFP, GSDMD-EGFP, DFNA5-EGFP and DFNA5-D270E-EGFP cell lines were generated by transfecting 293T cells with pEGFPN1 alone or pEGFPN1-GSDMD, pEGFPN1-DFNA5 or pEGFPN1-DFNA5-D270E plasmids followed by multiple cell sorting over a period of one month by flow cytometry.

### LDH release assay

Pyroptosis and necrosis were quantitated by assaying the activity of LDH released into cell culture supernatants after various treatments using the CytoTox96 LDH release kit (Promega) according to the manufacturer's protocol. The LDH activity in the culture supernatant was expressed as a percentage of total LDH in the cell lysate.

### siRNA knockdown

Knockdown of DFNA5 was performed by transfection of siRNA oligonucleotides targeting mouse DFNA5 (Dharmacon, Catalogue No. L-041196-01). Scrambled siRNA (control) or DFNA5-specific siRNA was transfected into immortalized Casp-1/11^−/−^ BMDMs using Lipofectamine 2,000 (Invitrogen) according to the manufacturer's protocol. Forty-eight hours after transfection cells were infected with VSV-GFP (Indiana strain) in OPTI-MEMI medium. The culture supernatants and cells were collected at indicated times for analysis.

### Immunoblot analysis

Cells were lysed in buffer containing 50 mM Tris, pH 7.5, 150 mM NaCl, 1 mM EDTA, 0.1% NP-40 and protease inhibitors. Cell lysates were fractionated by SDS–polyacrylamide gel electrophoresis (SDS–PAGE) and then transferred to PVDF membranes (Bio-Rad). In some experiments, cell culture supernatants were precipitated by methanol/chloroform as described previously^24^,^59^. Briefly, the culture supernatants were precipitated by the addition of an equal volume of methanol and 0.25 volumes of chloroform to each sample, and centrifuged at 14,000 r.p.m. in an Eppendorf tabletop 5417R refrigerated microcentrifuge. The upper phase was discarded and 500 μl methanol was added to the interphase of each sample. The samples were centrifuged for 10 min at 14,000 r.p.m. and the resulting protein pellets were dried at room temperature, resuspended in Laemmli buffer and boiled for 5 min at 99 °C. The resuspended proteins were fractionated on 12.5% SDS-polyacrylamide gels followed by electroblotting onto PVDF membranes. Blots were probed with appropriate antibodies. Full-size scans of immunoblots are shown in Supplementary Figs 12–24.

### *In vitro* caspase cleavage assay

Bacterially expressed caspases with C-terminal His6 tags were purified on Talon affinity resins and then eluted with 250 mM imidazole in CHAPS buffer (20 mM HEPES, pH 7.0, 10 mM KCl, 0.1% CHAPS). Bacterially expressed WT DFNA5, DFNA5-D270E and GSDMD with N-terminal His6-T7 tags were purified on Talon affinity resins and incubated at 37 °C for 40 min with purified caspases in CHAPS buffer in a total 20-μl reaction. The cleavage products were analysed by SDS–PAGE and visualized by western blotting or Coomassie staining.

### *In vitro* assay of DFNA5 cleavage by the apoptosome

293T-DFNA5, 293T-DFNA5-D270E, HEPG2 and casp-1/11-dKO cells were collected by centrifugation at 600*g* for 10 min. The cell pellets were washed twice with ice-cold phosphate-buffered saline (pH 7.4) and resuspended with 3 volumes of buffer A (250 mM sucrose, 20 mM HEPES, 10 mM KCl, 1.5 mM MgCl_2_, 1 mM EDTA, 1 mM EGTA, 1 mM dithiothreitol, 0.1 mM phenylmethylsulfonyl fluoride, pH 7.5) and left on ice for 15 min. The cells were lysed by syringing (30 × ) with a 26 G needle. The lysates were centrifuged at 14,000 r.p.m. for 10 min at 4 °C in a refrigerated eppendorf 5417R centrifuge. The supernatants were collected and further centrifuged at 100,000*g* for 30 min at 4 °C, and the resulting supernatants (designated S100 lysates) were used for *in vitro* assay of DFNA5 cleavage. To activate the Apaf-1 apoptosome, the S100 lysates were incubated with purified bovine cytochrome c (15 μg ml^−1^) for different periods of time at 37 °C in 20 μl reactions. After the incubation periods the reactions were stopped by adding equal volumes of SDS-sample buffer and analysed by SDS–PAGE and visualized by western blotting with appropriate antibodies.

### Cell fractionation for membrane localization

These experiments were performed as described recently^11^ with some modifications. Cell pellets were suspended in 3 volumes of buffer A and left on ice for 15 min before lysing by syringing through a 26 G needle (30 × ). The cell lysates were centrifuged at 800*g* for 10 min at 4 °C in a refrigerated eppendorf 5417R centrifuge and the resulting supernatants were divided into two aliquots. To one aliquot cytochrome c was added (15 μg ml^−1^) and incubated for 45 min at 37° C to activate caspase-3 and DFNA5, whereas the second aliquot was kept on ice. After the incubation period the two aliquots were centrifuged at 7,000*g* for 10 min and the resulting pellets containing heavy membranes (P7) were washed and then resuspended in the same volumes of buffer A. The supernatants (S7) were further centrifuged at 20,000*g* for 10 min and the resulting pellets containing light membranes (P20) were washed and then resuspended in the same volumes of buffer A. The supernatants (S20) were further centrifuged at 100,000*g* for 10 min. The resulting supernatants (S100) and pellets (P100) were collected and the pellets were then washed and resuspended in the same volumes of buffer A. All fractions were then analysed by SDS–PAGE followed by immunoblotting with DFNA5 antibody.

### Generation of *DFNA5* ^*−/−*^ mice

Heterozygous mice carrying *lacZ*-disrupted *DFNA5* gene (C57BL/6N-Dfna5^tm1b(KOMP)Wtsi^/Wtsi) were obtained from EMMA mouse repository (EMMA ID, EM:09924). These mice were generated from the KOMP/EUCOMM targeted ES cell resource using standard techniques as described previously^60^,^61^ and established on a C57BL/6 genetic background. *DFNA5* exon 5 was flanked by loxP sites, and subsequent Cre expression excised this exon resulting in a knockout reporter allele using a cell permeable HTN-Cre as described in^62^ (Supplementary Fig. 8). Mouse strains were maintained in specific pathogen-free conditions and the animal protocols were carried out in accordance with the guidelines set forth by Institutional Animal Care and Use Committee (Thomas Jefferson University).

### Structural modelling

A homology model of DFNA5 was generated using the SWISS-MODEL server^27^. Structural homologues were identified in the RCSB database using the DALI server^30^. Structural superimpositions were carried out in Coot^63^ and Chimera^64^. All ribbon diagrams presented in the paper were prepared using the program Pymol (DeLano, W.L. The PyMOL Molecular Graphics System, Version 1.8 Schrödinger, LLC. (2002)). The schematic helical representation of DNFA5 N-terminal helix was generated using the Helical Wheel Projections script (http://rzlab.ucr.edu/).

### Confocal microscopy

The 293T cells were seeded on 35 mm cover glass-bottom culture dishes and allowed to attach for 24 h. Cells were transfected with constructs for different DFNA5 mutants for 18–24 h using Lipofectamine 2,000 and then stained with Hoechst 33,342 for 30 min. Cells were observed using a Nikon A1R resonant scanning confocal microscope (Bioimaging Shared Resource of the Kimmel Cancer Center (NCI 5 P30 CA-56036)).

### Statistics

Statistical analyses were made with Student's *t*-test.

### Data availability

The data that support the findings of this study are available from the corresponding author on reasonable request.

## Additional information

**How to cite this article:** Rogers, C. *et al*. Cleavage of DFNA5 by caspase-3 during apoptosis mediates progression to secondary necrotic/pyroptotic cell death. *Nat. Commun.* **8,** 14128 doi: 10.1038/ncomms14128 (2017).

**Publisher's note:** Springer Nature remains neutral with regard to jurisdictional claims in published maps and institutional affiliations.

## Supplementary Material

Supplementary Information Supplementary Figures

Supplementary Movie 1 Plasma membrane targeting of DFNA5-N fragment causes membrane ballooning. C-terminal EGFP-tagged DFNA5-N fragment (DFNA5-N F2A) was expressed in 293T and then observed by confocal microscopy. Shown is a 3D reconstruction of the confocal z stack images of the GFP channel (DFNA5, green) of the cells shown in Fig. 3d, middle panel

Supplementary Movie 2 Plasma membrane targeting of DFNA5-N fragment causes membrane ballooning. C-terminal EGFP-tagged DFNA5-N fragment (DFNA5-N F2A) was expressed in 293T and then observed by confocal microscopy. Shown is a cross-section segment (central slice) of the 3D reconstruction of the confocal z stack images of the two cells shown in video 1. Only the GFP (DFNA5) channel is shown

Supplementary Movie 3 Plasma membrane targeting of DFNA5-N fragment causes membrane ballooning. C-terminal EGFP-tagged DFNA5-N fragment (DFNA5-N F2A) was expressed in 293T and then observed by confocal microscopy. Shown is a cross-section segment (central slice) of the 3D reconstruction of the confocal z stack images of the two cells shown in video 1. All channels including GFP (DFNA5, green), Hoechst (nucleus, blue) and phase contrast (gray) channels are included.

Supplementary Movie 4 Time-lapse confocal microscopy imaging of DFNA5+/+ and DFNA5-/- macrophages undergoing etoposide-induced apoptosis (representative field 1). Image recording was initiated 90 minutes after treatment of DFNA5+/+ (upper) and DFNA5-/- (lower) macrophages with etoposide (150 μM) and continued at a rate of one image every two minutes for an additional 150 minutes.

Supplementary Movie 5 Time-lapse confocal microscopy imaging of DFNA5+/+ and DFNA5-/- macrophages undergoing etoposide-induced apoptosis (representative field 2). Image recording was initiated 90 minutes after treatment of DFNA5+/+ (upper) and DFNA5-/- (lower) macrophages with etoposide (150 μM) and continued at a rate of one image every two minutes for an additional 150 minutes.
