# Supplementary material for: Cleavage of DFNA5 by caspase-3 during apoptosis mediates progression to secondary necrotic/pyroptotic cell death
Source: Nat Commun. 2017 Jan 3;8:14128. doi: 10.1038/ncomms14128 (PMC5216131; doi:10.1038/ncomms14128)
Supplement: Supplementary Information — Supplementary Figures [file ncomms14128-s1.pdf]

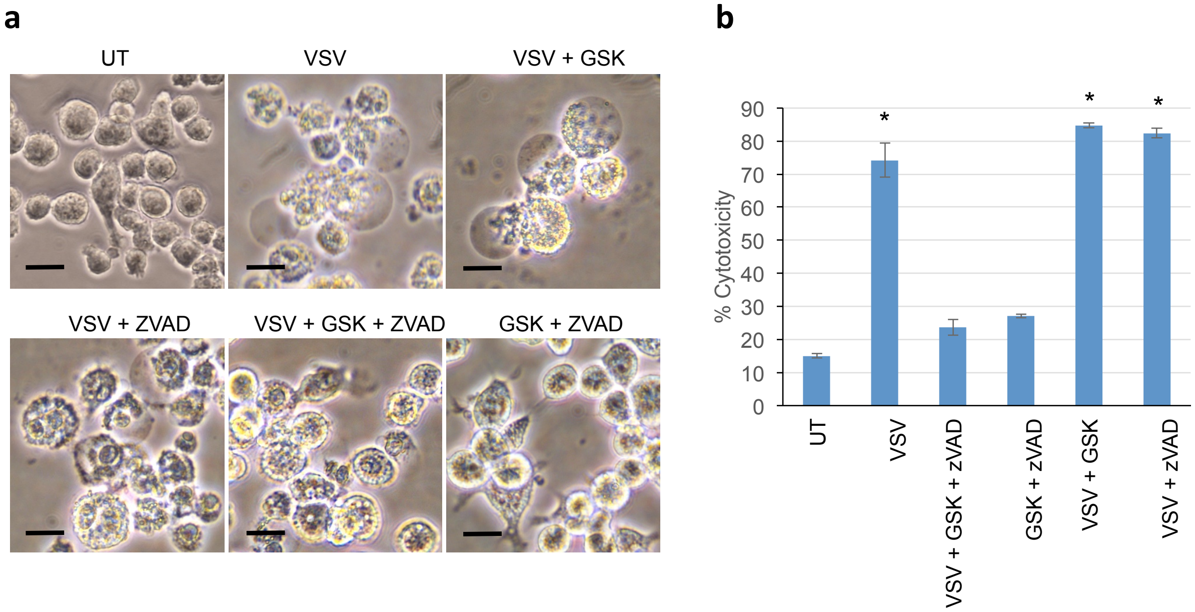

Supplementary Figure 1: VSV infection induces caspase-1/11-independent necrosis/pyroptosis in macrophages. (**a**) Microscopic images of immortalized casp-1/11-dKO macrophages infected with VSV (10 MOI, 16 h) in the absence or presence of zVAD-fmk (30 µM), GSK'872 (10 µM) or zVAD plus GSK'872, as indicated. Scale bar, 20 µm. (**b**) Cytotoxicity of VSV (10 MOI, 16 h) as measured by LDH release in the culture supernatants of VSV-infected casp-1/11 dKO macrophages in the absence or presence of zVAD-fmk, GSK'872 or zVAD plus GSK'872, as indicated. *, $p<0.001$.

a

```
                     *                                                  ** *
Human DFNA5  ---MFAKATRNFLREVD-ADGDLIAVSNLNDSDKLQLLSLVTKK-KRFWCWQRPKYQFLSLTLGDVLIEDQFPSPVVVES 75
mouse GSDMD  MPSAFEKVVKNVIKEVSGSRGDLIPVDSLRNSTSFRPYCLLNRKFSSSRFW-KPRYSCVNLSIKDIL-EPSAPEPEP-EC 77

Human DFNA5  DFVKYE-GKFAN-HVSG-TLETALGKVKLNLGGSSRVESQS-SFGTLRKQEVDLQQLIRD-SAERTI-NLRNPVLQQVLE 149
mouse GSDMD  -FGSFKVSDVVDGNIQGRVMLSGMGEGKIS-GGAAVSDSSSASMNVCILR-VT-QKTWETMQHERHLQQPENKILQQ-LR 152

Human DFNA5  GRNEVLCVLTQKITTMQKCVISEHMQVEEKCGGIVGIQTKTVQVSATEDGNVTKDSNVVLEIPAATTIAYGVIELYVKLD 229
mouse GSDMD  SRGDDLFVVTEVLQTKEEVQITE-VHSQEGSGQFT-LPG-ALCLKGEGKGHQSRK-KMV-TIPAGSILAFRVAQL-L-IG 225

Human DFNA5  GQFEFCLLRG-KQGGFENKKRIDSVYLDPLVFREFAFIDMPDAAHGISSQDGPLS-VLKQATLLLERNFH-PFAELPEPQ 306
mouse GSDMD  SKWDILLVSDEKQRTFEPSSG-DRKAVGQ---RHHG-LNVLAALCSIGKQLSLLSDGIDEEELIEAADFQGLYAEV-KAC 299

Human DFNA5  QTALSDIFQAVLFDDELLMVLEPVCDDLVSGLSPTVAVLGE-LKPRQQQDLVAFLQLVGCSLQGGCPGPEDAGS--KQLF 383
mouse GSDMD  SSELESL-EMEL-RQQILVNIGKILQDQPS-MEALEASLGQGLCSGGQVEPLD--GPAGCILE--CLVL-DSGELVPELA 371

Human DFNA5  MTAYFLVSALAEMPDSAAALLGTCCKLQII-P--TLC-HLLRALSD-DGVSDLEDPTLTPLKD--TERFGIVQRLFASAD 456
mouse GSDMD  APIFYLLGALAVLSETQQQLLAKALETTVLSKQLELVKHVLEQSTPWQEQSSVSLPTVL-LGDCWDEK-NPTWVLLEECG 449

Human DFNA5  ISLERLKSSVKAVILKDSKVFPL-LLCITLNGLCALGREHS 496
mouse GSDMD  LRLQ-VESP-Q-VHWEPTSLIPTSALYASLFLLSSLGQKPC 487
```

b

```
                     *                                                  ** *
Human DFNA5  ---MFAKATRNFLREVDADGDLIAVSNLNDSDKLQLLSLVTKK-KRFWCWQRPKYQFLSLTLGDVLIEDQFPSPVVVES- 75
Human GSDMD  MGSAFERVVRRVVQELDHGGEFIPVTSLQSSTGFQPYCLVVRKPSSSWFW-KPRYKCVNLSIKDIL-EPDAAEPDVQRGR 78

Human DFNA5  DFVKYEGKFANHVSGTLETAL-GKVKLNLGGSSRVESQSSFGTLRKQEVDLQQLIRDSAERTINL-RNPVLQQVLEGRNE 153
Human GSDMD  SFHFYDA-MDGQIQGSVELAAPGQAKI-AGGAAVSDSSSTSMNVYSLSVDPNTWQTLLHERHLRQPEHKVLQQ-LRSRGD 155

Human DFNA5  VLCVLTQKITTMQKCVISEHMQVEEKCG-GIVGIQTKTVQVSATEDGNVTKDSNVVLEIPAATTIAYGVIELYVKLDGQF 232
Human GSDMD  NVYVVTEVLQT-QK-EV-EVTRTHKREGSGRFSLPGATC-LQGEGQGHLSQKKTVT--IPSGSTLAFRVAQL-V-IDSDL 227

Human DFNA5  EFCLLRG-KQGGFENKKRIDSVYLDPLVFREF-AFIDMPDAAHGISSQDGPLSVLKQATLLLERNFHPF-AELPEPQQTA 309
Human GSDMD  DVLLFPDKKQRTFQPPATGHKRSTSEGAWPQLPSGLSMMRCLHNFLT-DG-VPA-EGA-FT-E-DFQGLRAEV-ETISKE 300

Human DFNA5  LSDIFQAVLFDDELLMVLEPVC-DDL-VSGLSPTVAVLGE-LKPRQQQDLVAFLQLVGC-SLQGGCPGPEDAGSKQLFMT 385
Human GSDMD  L-ELLDRELCQ-LLLEGLEGVLRDQLALRALEEALEQ-GQSLGPVEPLDGPAGAVLE-CLVLSSGMLVPELA----I-PV 371

Human DFNA5  AYFLVSALAEMPDSAAALLGTCCKLQ-IIPTLCHLLRALSDDGVSDLEDPT--LTP-LKDTERFGIVQRLFASAD-ISLE 460
Human GSDMD  VY-LLGALTMLSETQHKLLAEALESQTLLGPL-ELVGSLLEQSAPWQERSTMSLPPGLLGNS-WGEGAPAWVLLDECGLE 448

Human DFNA5  RLKSSVKAVILK-DSKVFPLLLCITLNGLCALGRE-HS 496
Human GSDMD  -LGEDTPHVCWEPQAQGRMCALYASLALLSGLSQEPH- 484
```

Supplementary Figure 2: Co-linear alignments of human DFNA5 and mouse GSDMD (a) or human DFNA5 and human GSDMD (b). The caspase cleavage sites in both DFNA5 and GSDMD are boxed red. The critical N-terminal phenylalanine and lysines in DFNA5 involved in membrane targeting and insertion are indicated by asterisks.

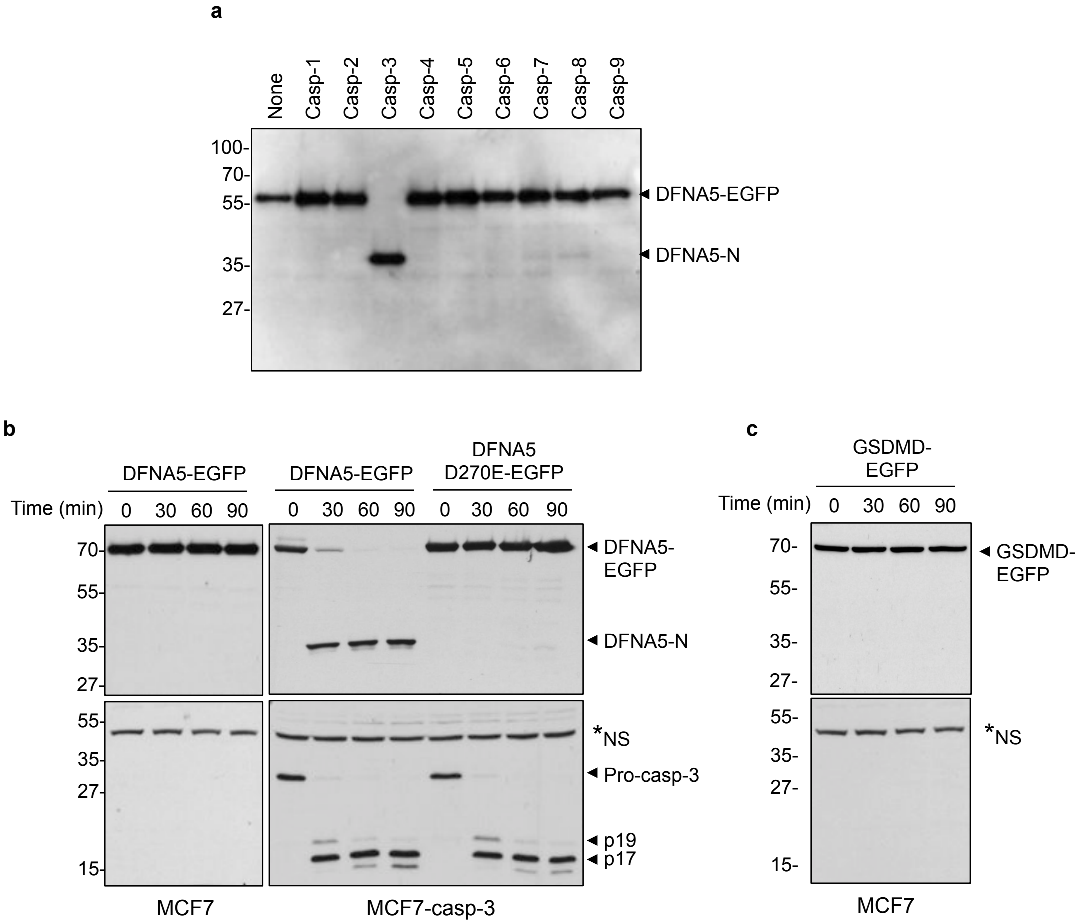

Supplementary Figure 3: Caspase-3 is responsible for DFNA5 cleavage. (**a**) Immunoblot of purified N-terminal T7-tagged WT DFNA5 incubated without (none) or with the indicated recombinant human caspases for 45 min at 37°C. The blot was probed with anti-T7 antibody. (**b**) Immunoblots of S100 lysates from MCF7 cells transfected with DFNA5-EGFP plasmid (left panel) or MCF7-casp-3 cells transfected with DFNA5-EGFP or DFNA5-D270E-EGFP plasmid (right panel), and then stimulated with cytochrome c for the indicated times at 37°C. The blots were probed with anti-DFNA5 (upper) or anti-caspase-3 (lower). (**c**) Immunoblots of S100 lysates from MCF7 cells transfected with GSDMD-EGFP plasmid and then stimulated with cytochrome c for the indicated times at 37°C. The blots were probed with anti-GSDMD (upper) or anti-caspase-3 (lower). Asterisks in **b**, **c** indicate non-specific bands, which serve as internal loading controls (NS).

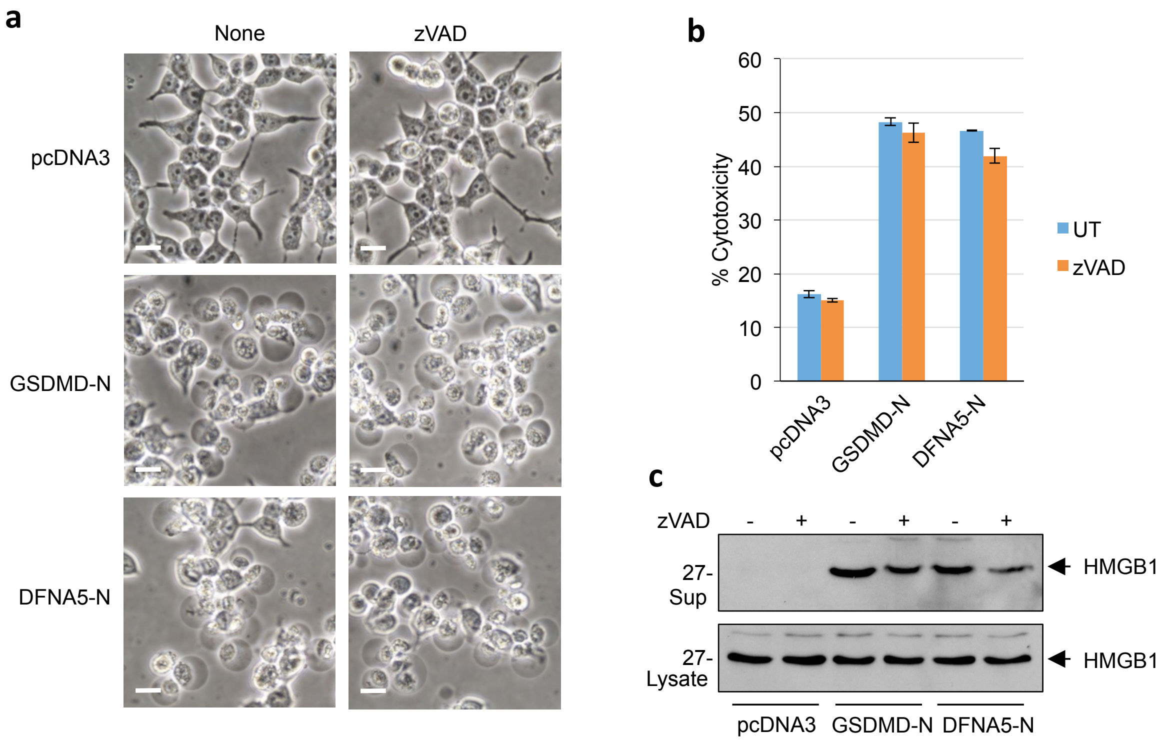

Supplementary Figure 4: DFNA5 and GSDMD-N fragments kill cells independent of caspase activation (**a**) Microscopic images of 293T cells transfected with empty vector (pcDNA3) or expression constructs for DFNA5-N or GSDMD-N fragment in the absence (None) or presence of zVAD (30 mM) for 24 h as indicated . Scale bar, 20 μm. (**b**) Cytotoxicity of DFNA5-N and GSDMD-N as measured by LDH release in the culture supernatants of 293T cells transfected with the indicated expression constructs in the absence (UT) or presence of zVAD as in **a**. (**c**) Immunoblots of HMGB1 in the culture supernatants (sup) and lysates of the cells described in **a** and **b**.

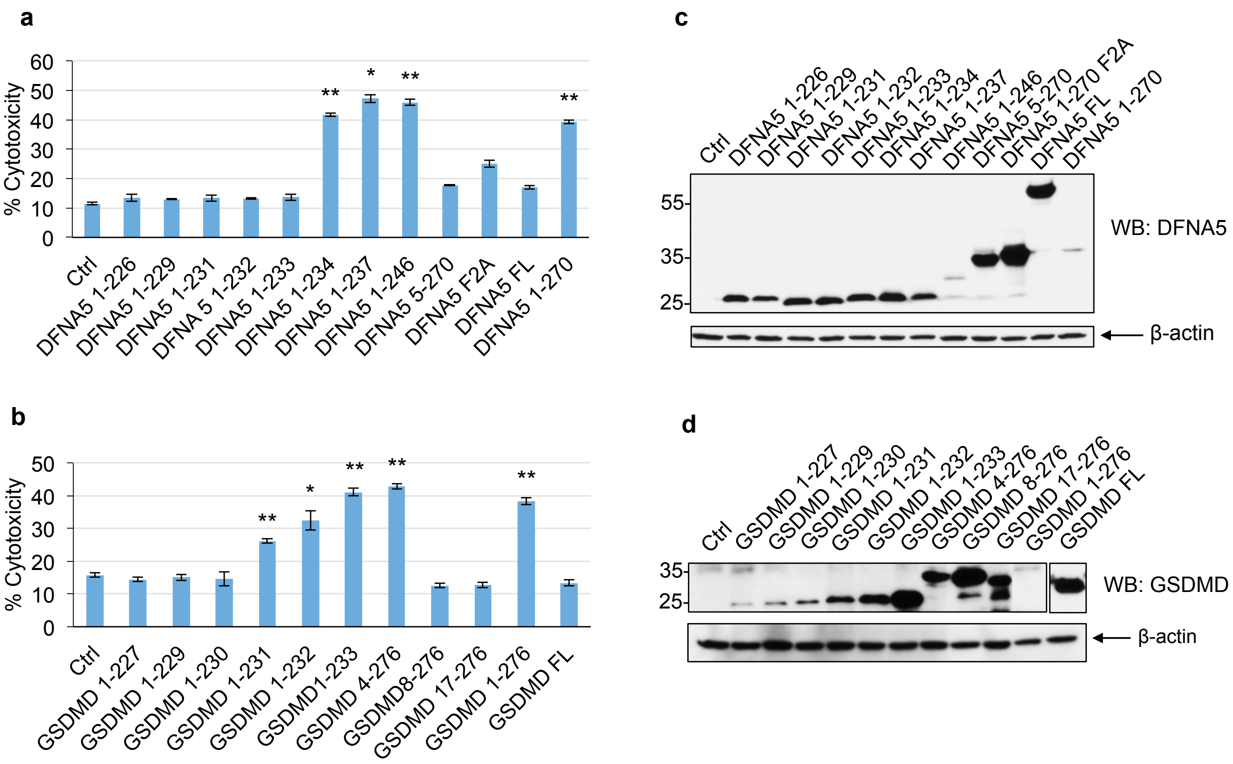

Supplementary Figure 5: Molecular determinants of the necrotic/pyroptotic activity of the DFNA5-N and GSDMD-N fragments. (**a-b**) Cytotoxicity of mutant DFNA5-N (**a**) (*, p<0.0001; **, p<0.00001) and GSDMD-N (**b**) (*, p<0.01; **, p<0.0001) fragments as measured by LDH release in the culture supernatants of 293T cells transfected with the indicated expression constructs for these mutants. (**c-d**) Immunoblots showing the expression of the mutant DFNA5-N and GSDMD-N fragments analyzed in **a** and **b**, respectively.

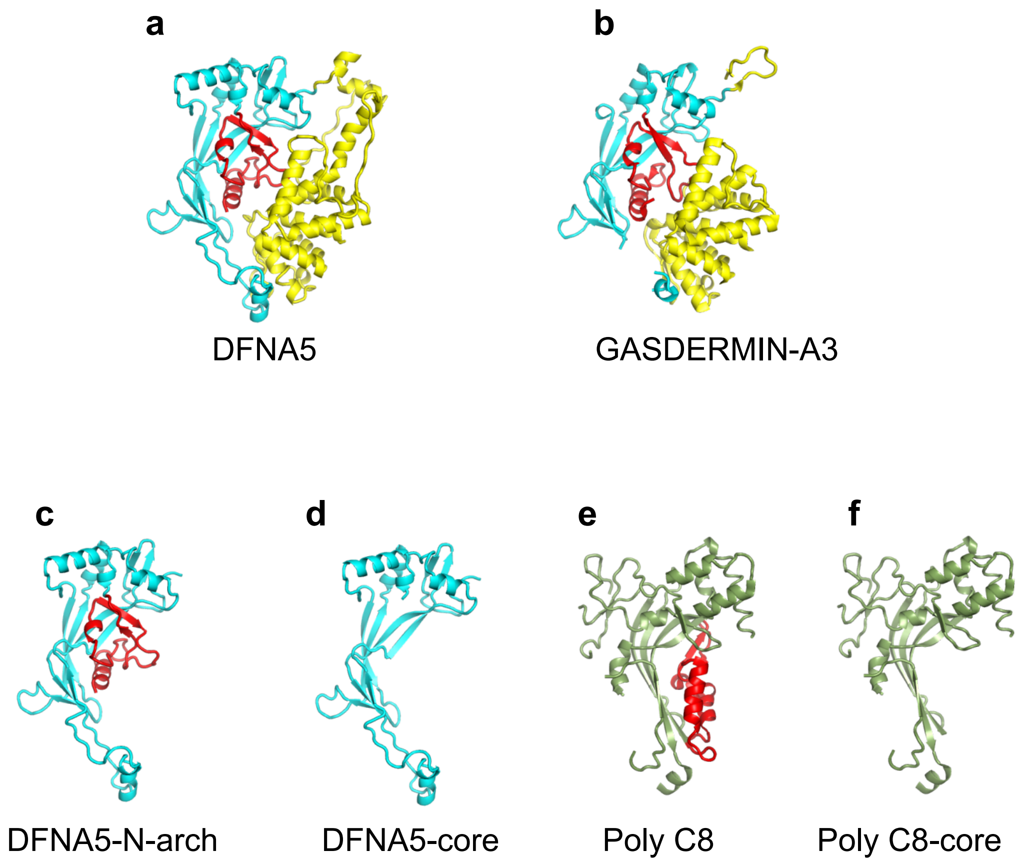

Supplementary Figure 6: Structural modeling of DFNA5. Ribbon diagrams of a homology model of DFNA-5 (**a**) based on the crystal structure of GSDMA3 (pdb 5b5r) (**b**). (**c**) DFNA5 N-terminal arch generated upon caspase-3 cleavage. (**d**) Structure of DFNA5 core used to identify structural homologs using the DALI server (PMID: 20457744) (Ref 30). (**e**) Crystal structure of the complement component C8a protein (Poly-C8) (PMID:17872444) (ref 29) that shares a superimposable fold with DFNA5 (rmsd ~5.8 Å) despite minimal sequence identity (~5.1%). (**f**) Structure of Poly-C8 core lacking the region involved in membrane penetration.

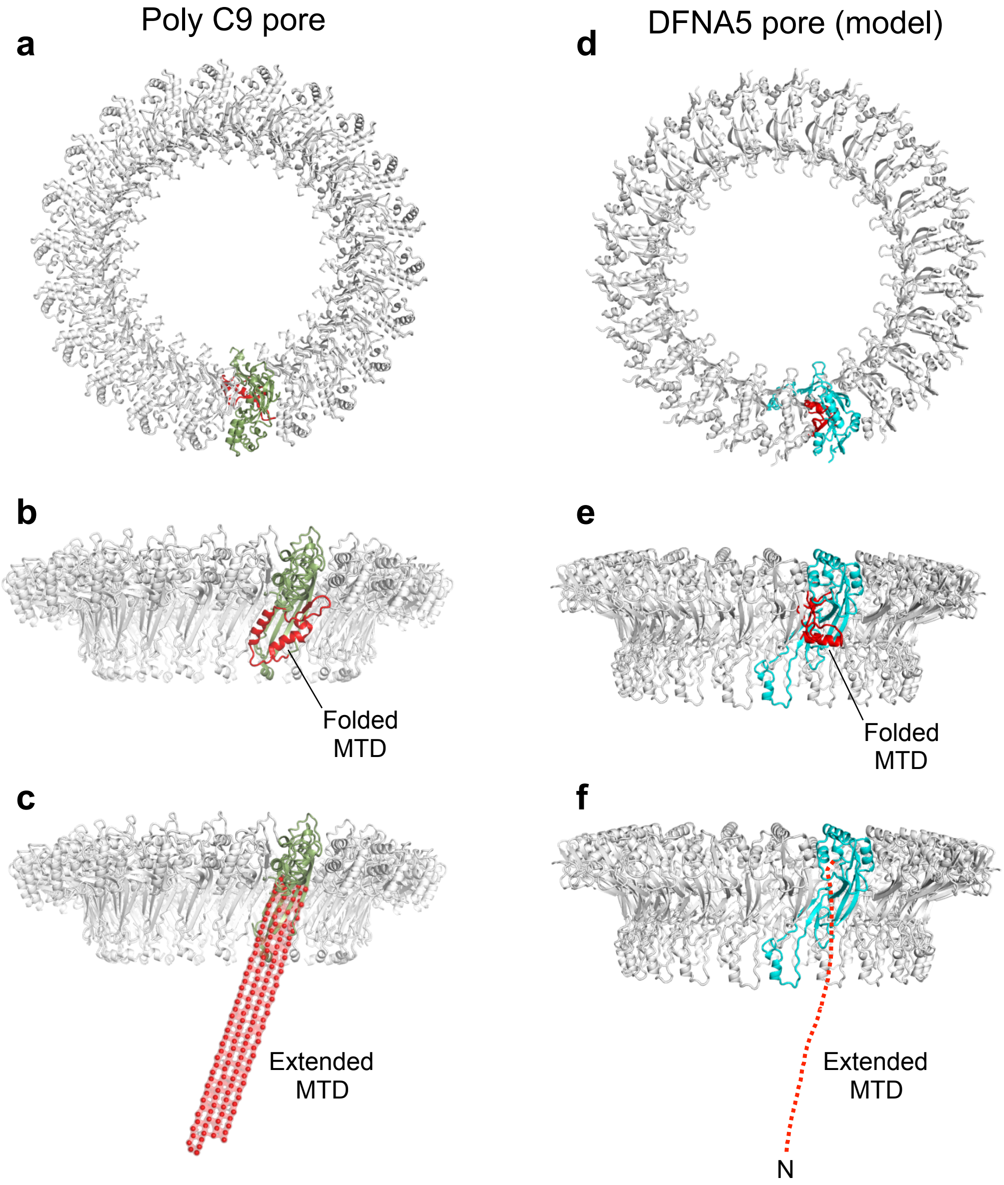

Supplementary Figure 7. A model of oligomeric DFNA5. (**a-c**) Pseudo-atomic model of the 22-fold symmetric ring formed by Poly-C9 upon membrane insertion (PMID:26841934) (ref 31). The membrane penetration moiety is closed and folded in panel **b** and extended in panel **c** (PMID:26841934) (ref 31). (**d-f**) Model of DFNA5 pore generated by superimposing DFNA5-core onto the structure of oligomeric Poly-C9. The putative N-terminal MTD is shown in its closed conformation in panel **e** and in a putative extended conformation in **f**.

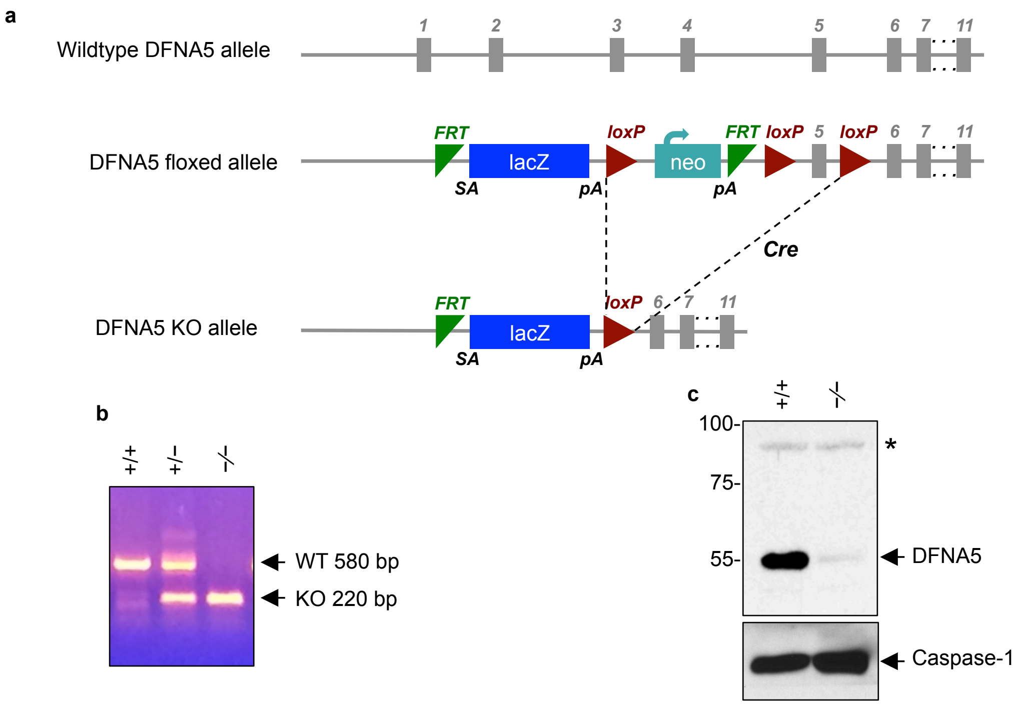

Supplementary Figure 8: **Generation of DFNA5 knockout mice.** (**a**) The DFNA5 floxed allele contains an IRES:*lacZ* trapping cassette and a floxed promoter-driven *neo* cassette inserted into the intronic sequence between exon 4 and 5 of the DFNA5 gene. Cre deletes the promoter-driven selection cassette and floxed exon of the DFNA5 allele to generate a *lacZ*-tagged allele which disrupts DFNA5 transcript. (**b**) genotyping of $DFNA5^{+/+}$, $DFNA5^{+/-}$and $DFNA5^{-/-}$ littermates shows deletion of exon 5 in $DFNA5^{-/-}$ mice. (**c**) Immunoblots of macrophage S100 lysates probed with anti-DFNA5 (upper) or anti-caspase-1 (lower) antibody show absence of DFNA5 in lysates from $DFNA5^{-/-}$ macrophages. The faint anti-DFNA5 antibody-reactive band co-migrating with the DFNA5 band represents a non-specific band some times seen in some immunoblots. Asterisk indicates a non-specific band serving as an internal loading control.

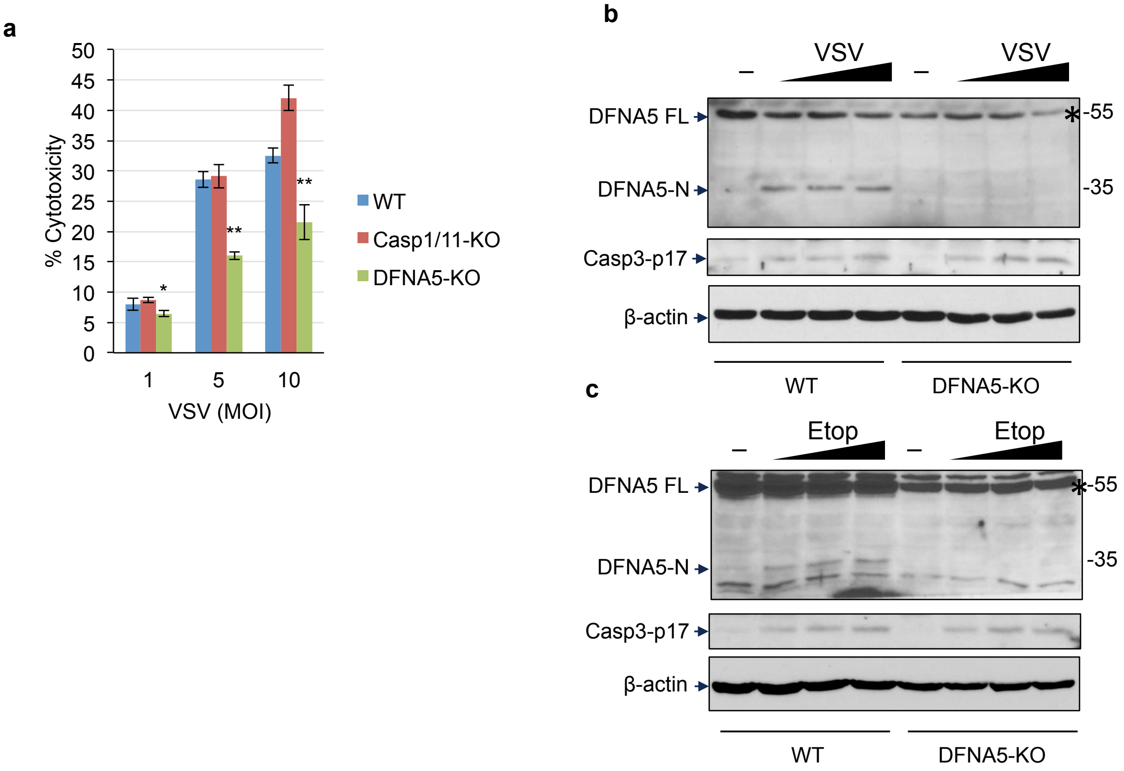

Supplementary Figure 9: (**a**) Cytotoxicity of VSV as measured by LDH release in the culture supernatants of immortalized WT, DFNA5-KO and casp-1/11 dKO BMDMs infected with VSV for 8 h. *. P<0.05, **p<0.005. (**b**, **c**) VSV and etoposide induce processing of DFNA5. (upper) Immunoblots of DFNA5, processed caspase-3 p17 and β-actin in cell lysates from WT or DFNA5-KO macrophages uninfected (-) or VSV-infected (1, 5, 10 MOI, 8h) (**b**) or untreated (-) or etoposide-treated (37.5, 75, 150 µM, 8h)(**c**). Asterisks, the faint anti-DFNA5 antibody-reactive band co-migrating with the DFNA5 band represents a non-specific band some times seen in some immunoblots.

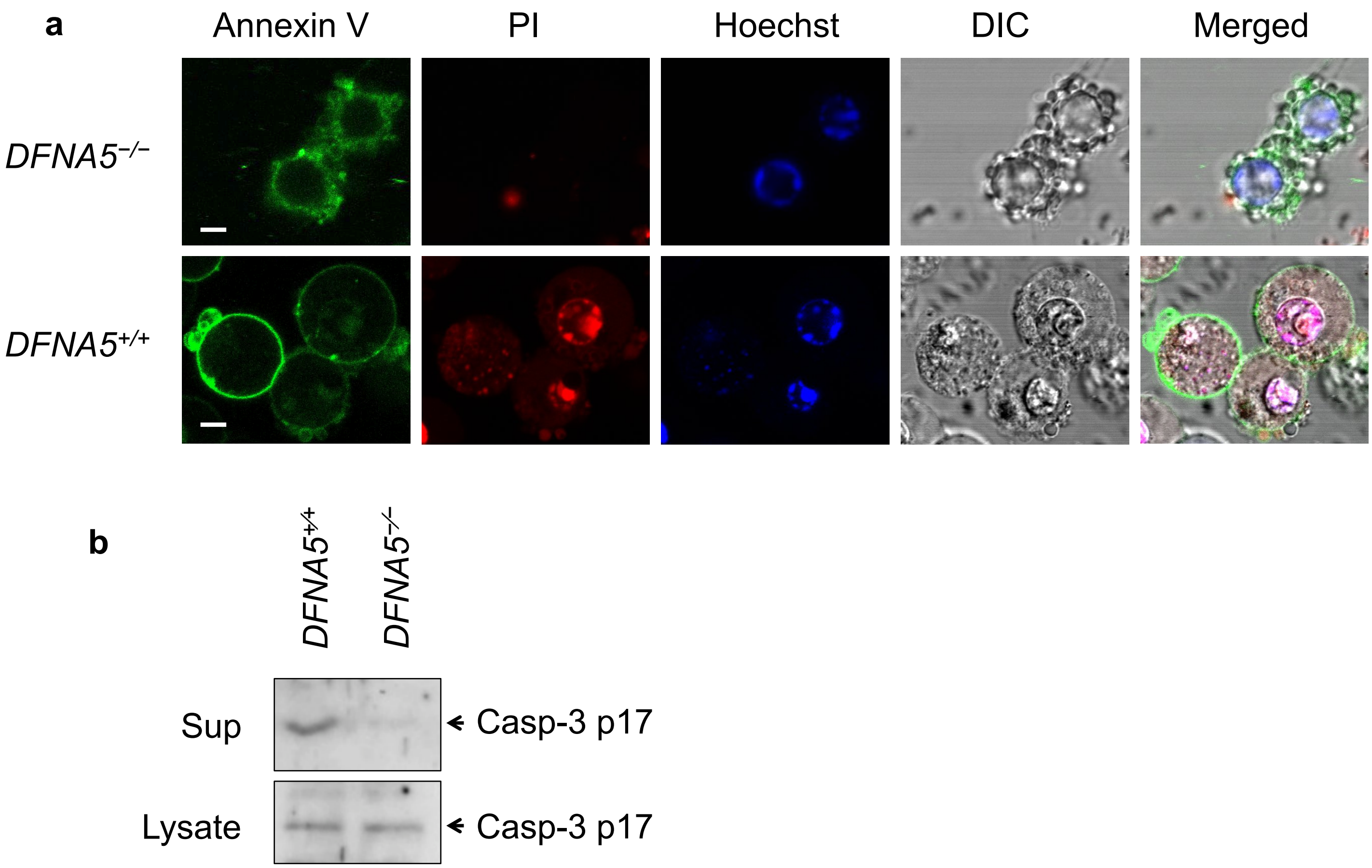

Supplementary Figure 10: (a) Confocal live-cell imaging of etoposide-treated *DFNA5*$^{+/+}$ and *DFNA5*$^{-/-}$ macrophages. Immortalized *DFNA5*$^{+/+}$ and *DFNA5*$^{-/-}$ macrophages were treated with etoposide (150 µM) for 4 h in 10 mm glass-bottom dishes. Cells were then stained with Annexin V-FITC, PI and Hoechst stains followed by confocal microscopy. Scale bar, 10 µm. (**b**) Immunoblot of caspase-3 p17 fragment in apoptotic culture supernatants (sup) of *DFNA5*$^{+/+}$ and *DFNA5*$^{-/-}$ macrophages. The lower panel shows caspase-3 p17 in the cell lysates.

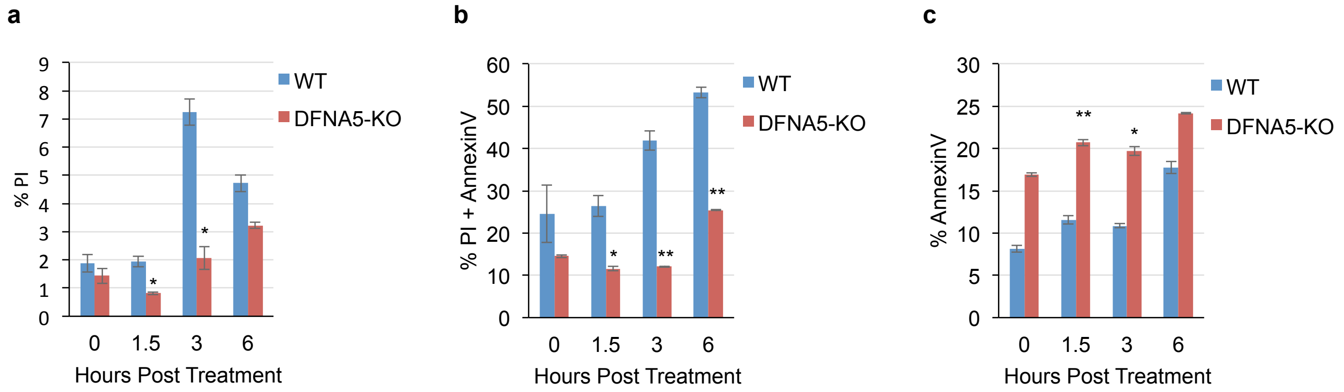

Supplementary Figure 11: Time-course analysis of etoposide-induced cell death in WT and DFNA5-KO macrophages by Annexin V-FITC/PI staining. Immortalized WT and DFNA5 macrophages were treated with etoposide (150 µM) in 6-well plates ($2x10^6$ cells/well) for the indicated times and then resuspended in PBS by repeated pipetting, followed by staining with Annexin V-FITC and PI stains and flow cytometric analysis. (**a**) PI-positive cells (%). * $p<0.01$. (**b**) Annexin V-FITC/PI–double positive cells (%). * $p<0.005$. ** $p<0.0005$. (**c**) Annexin V-FITC-positive cells (%). * $p<0.01$. ** $p<0.005$. FITC, fluorescein isothiocyanate; PI, propidium iodide.

Figure 1a.

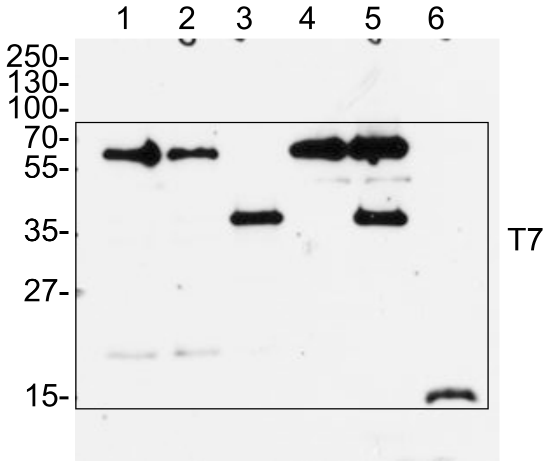

Figure 1d.

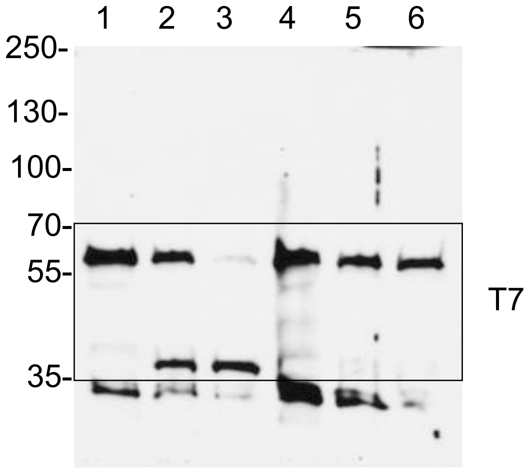

Supplementary Figure 12: Full-sized scans of Western blots in Figure 1a, d.

Figure 2a.

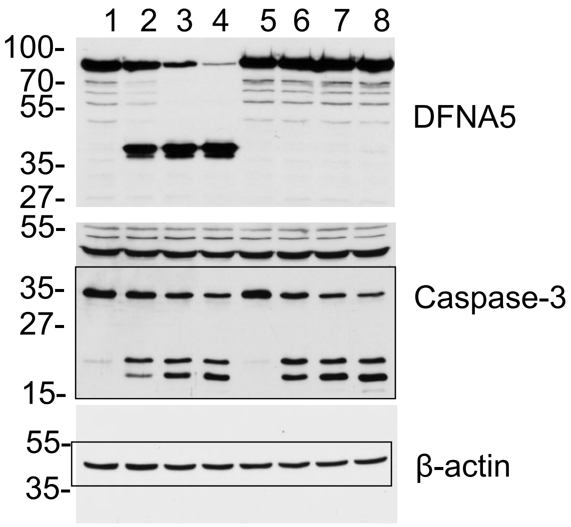

Figure 2b.

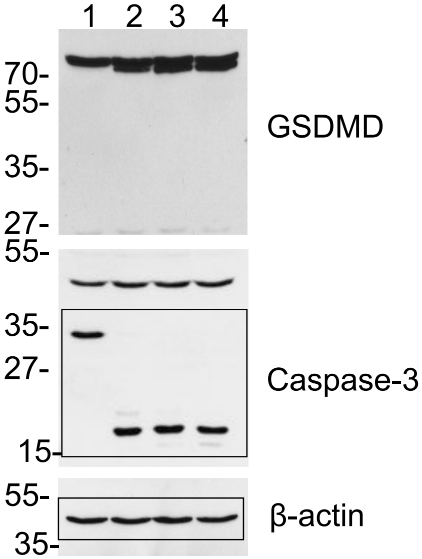

Figure 2c.

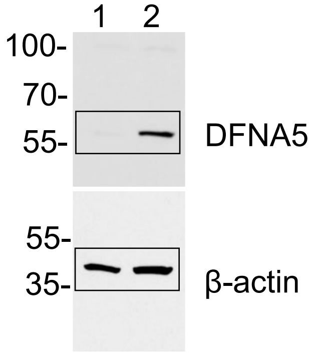

Figure 2d.

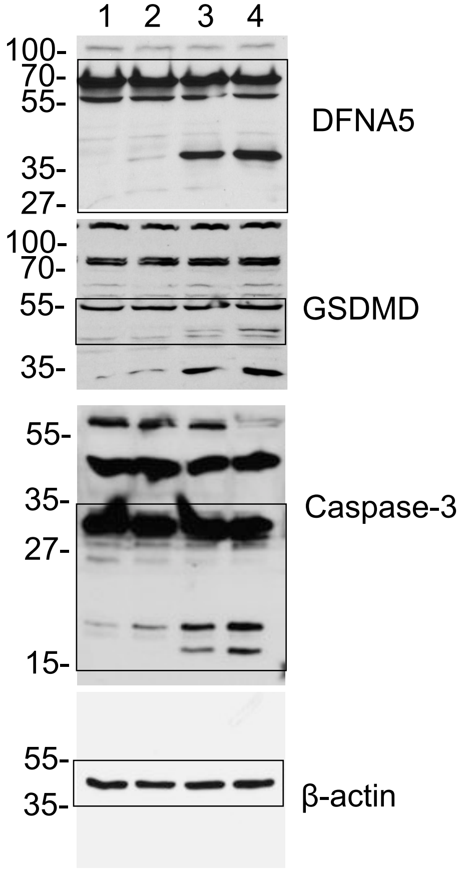

Figure 2e.

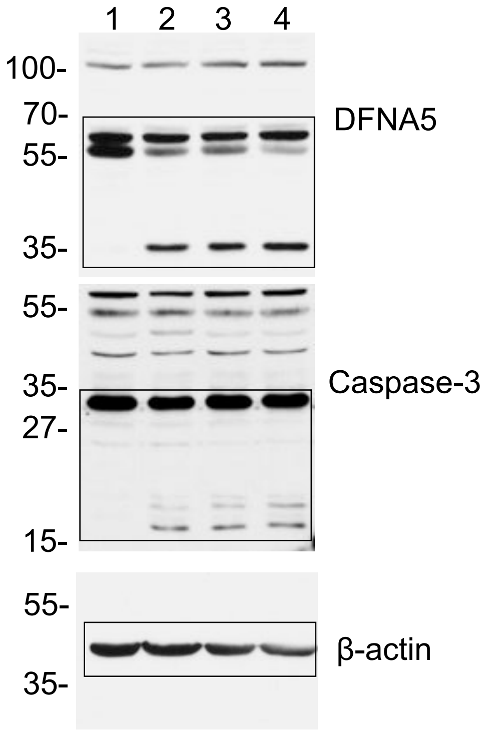

Supplementary Figure 13: Full-sized scans of Western blots in Figure 2a-e.

Figure 3c.

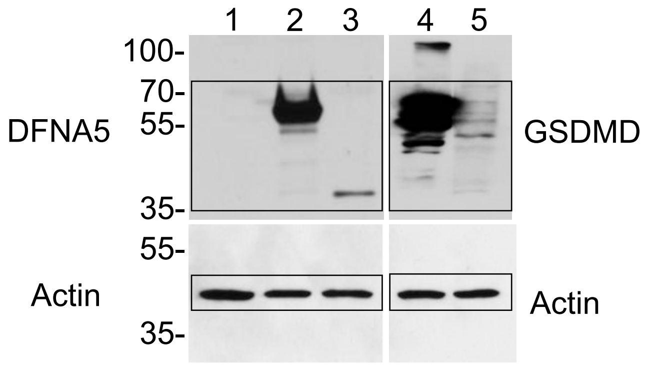

Figure 3e.

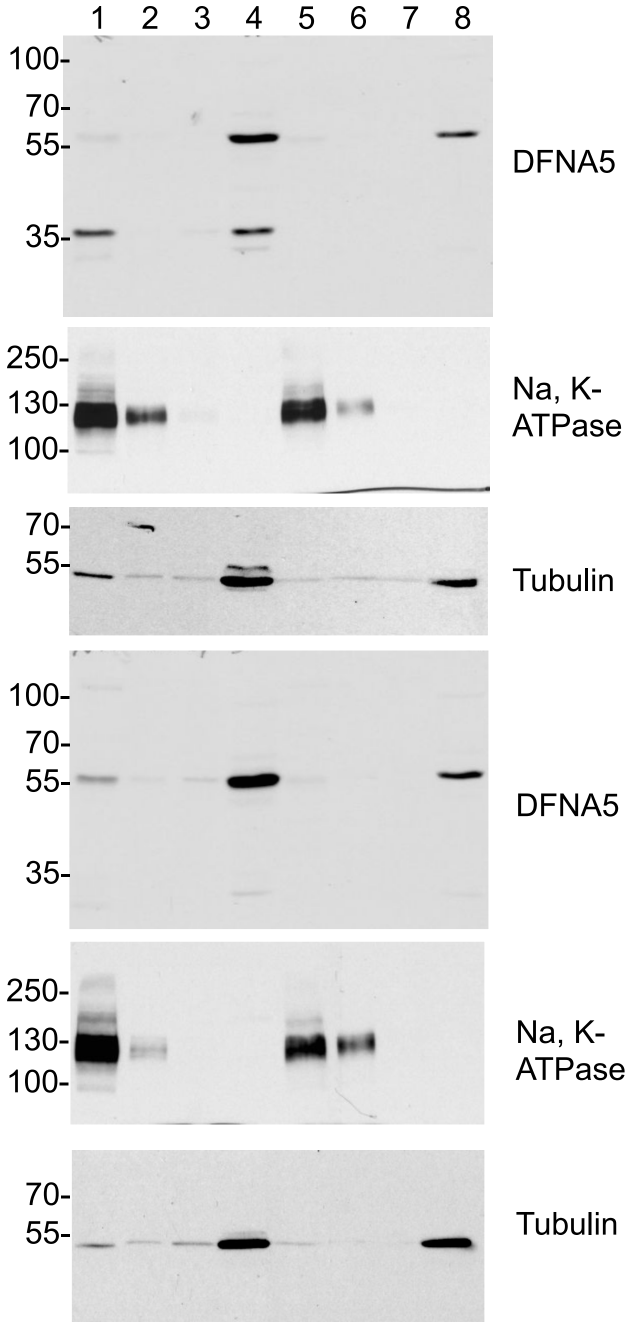

Figure 3f.

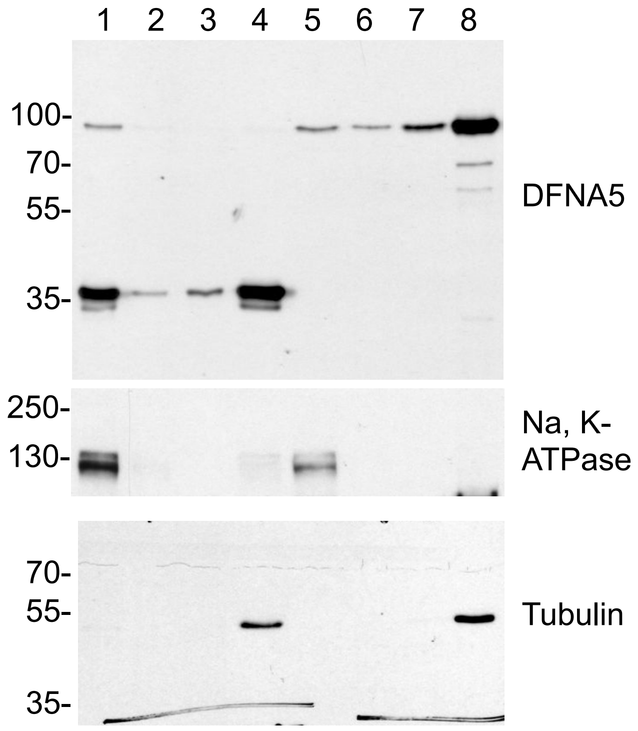

Supplementary Figure 14: full-sized scans of Western blots in figure 3c, e, f.

Figure 4h.

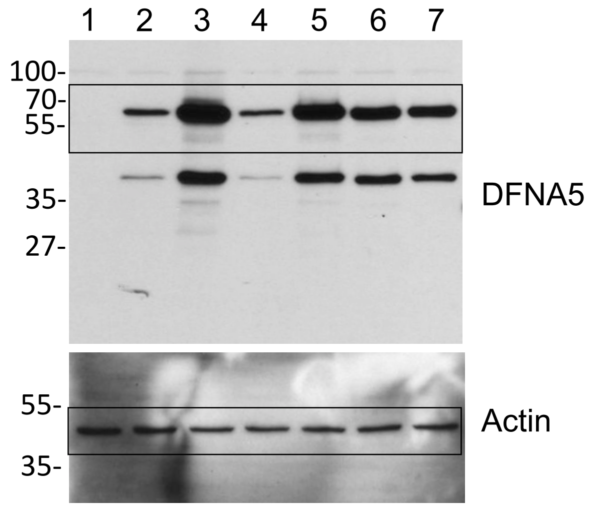

Supplementary Figure 15: full-sized scans of Western blots in figure 4h.

Figure 5a.

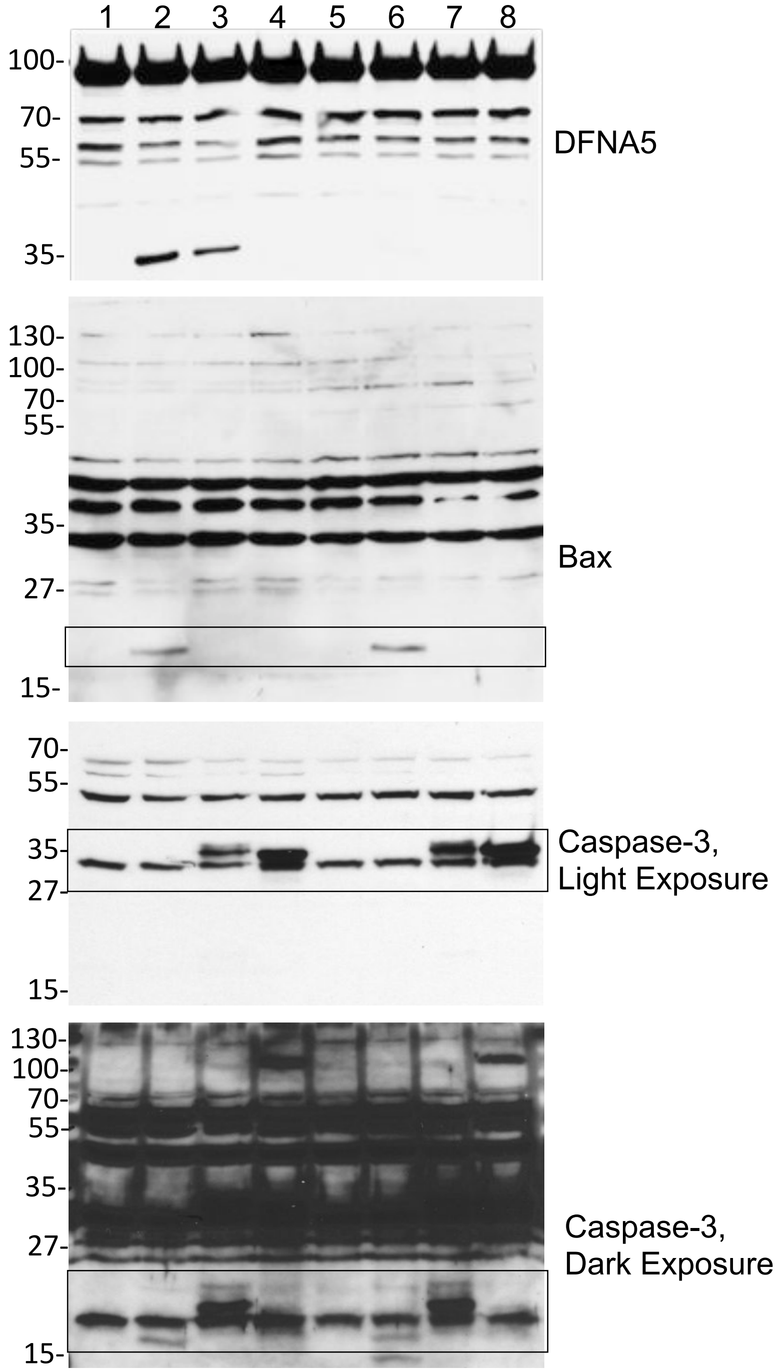

Supplementary Figure 16: full-sized scans of Western blots in figure 5.a

Figure 6a.

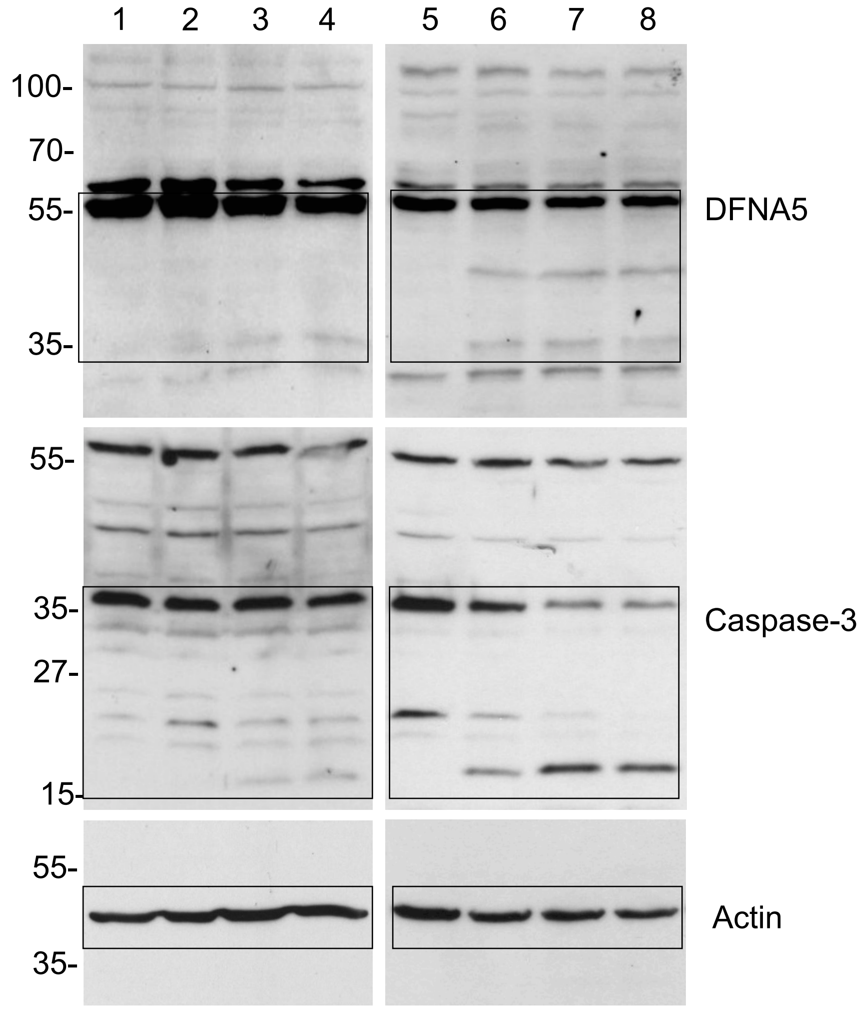

Figure 6d.

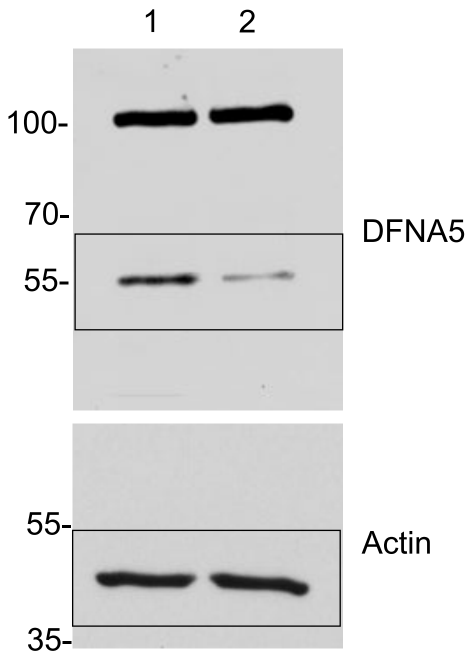

Supplementary Figure 17: full-sized scans of Western blots in figure 6a,d.

Figure 7b.

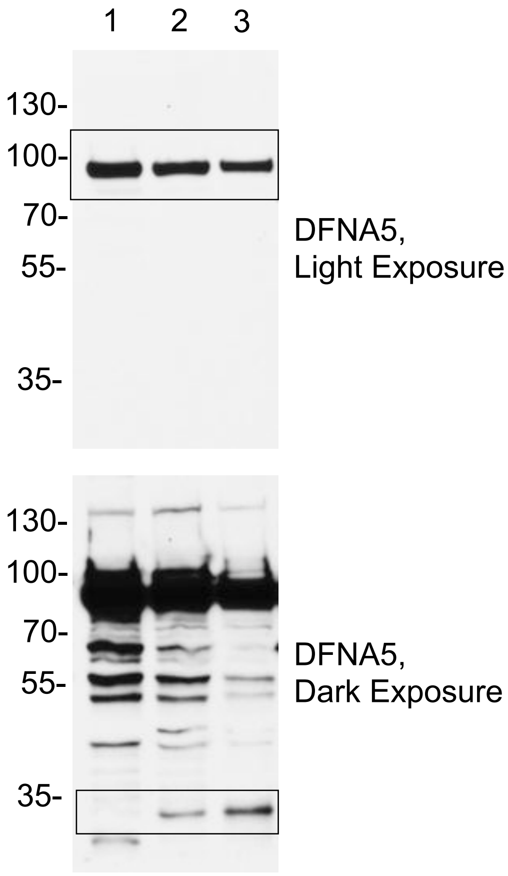

Figure 7e.

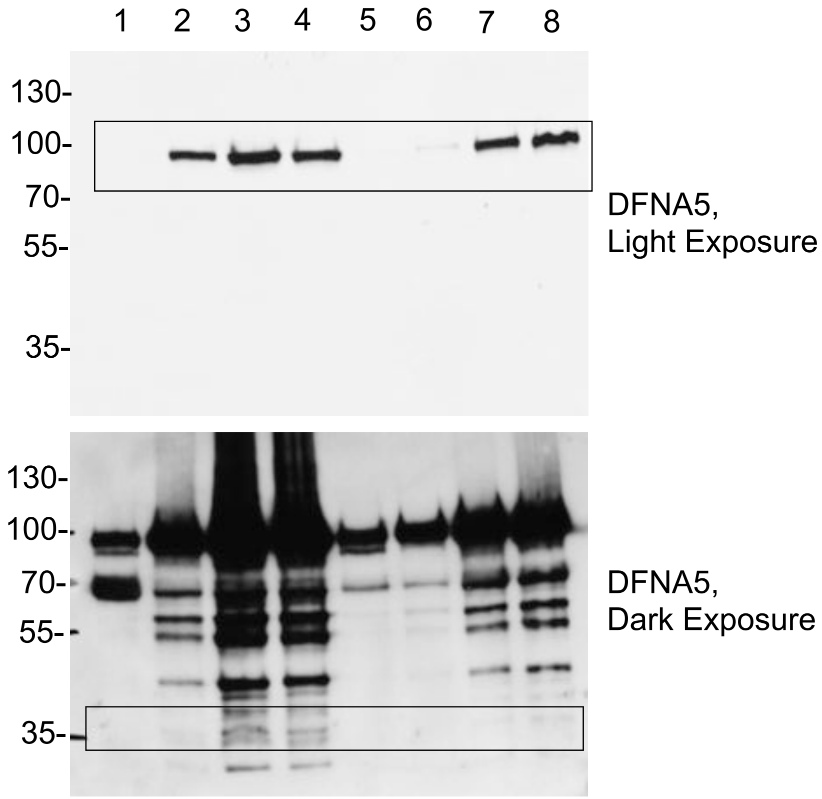

Supplementary Figure 18: Full-sized scans of Western blots in Figure 7b, e.

Figure 8a.

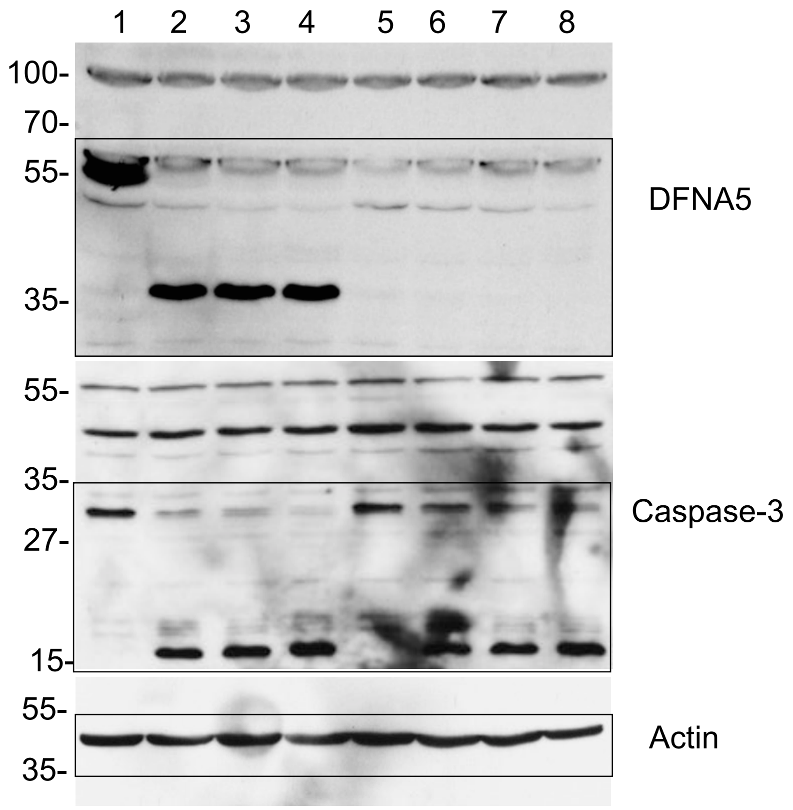

Supplementary Figure 19: Full-sized scans of Western blots in Figure 8a.

Supplementary Figure 4c.

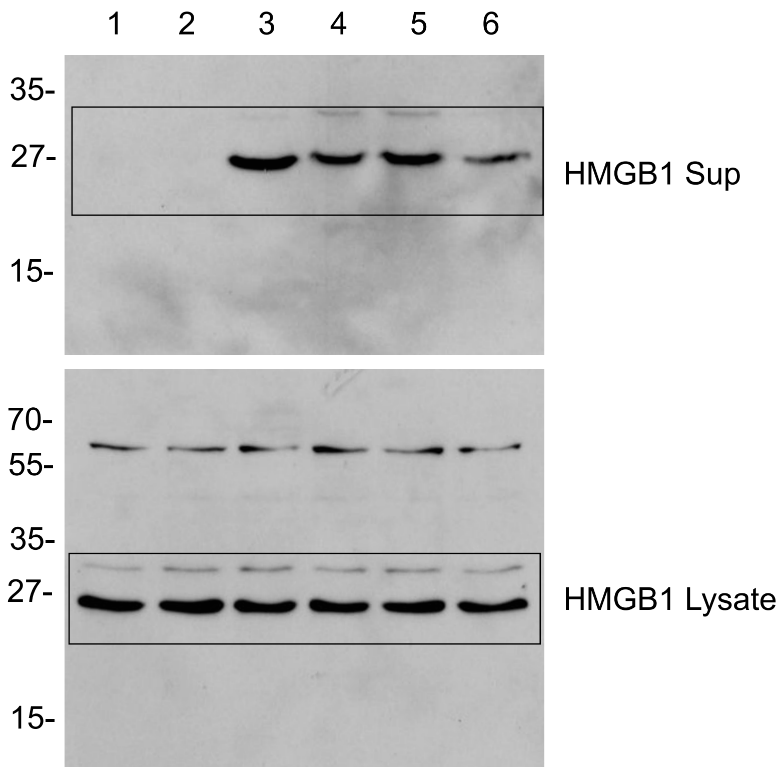

Supplementary Figure 20: Full-sized scans of Western blots in Supplementary Figure 4c.

Supplementary Figure 5c.

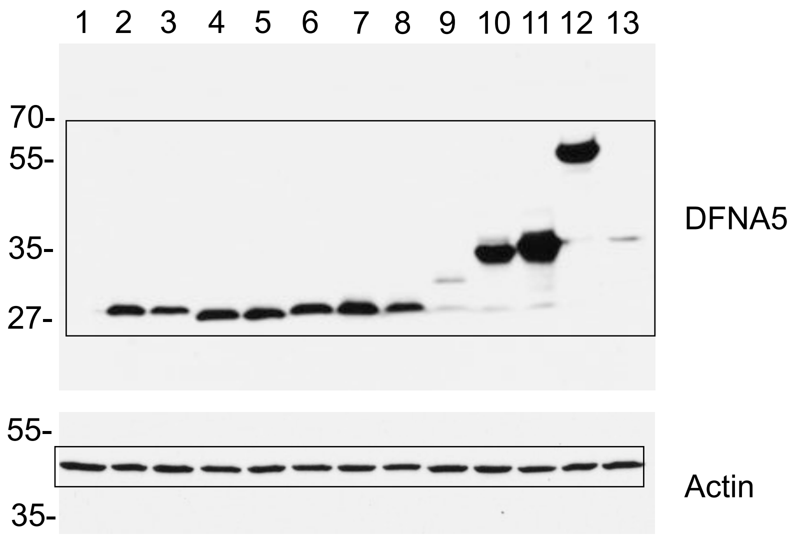

Supplementary Figure 5d.

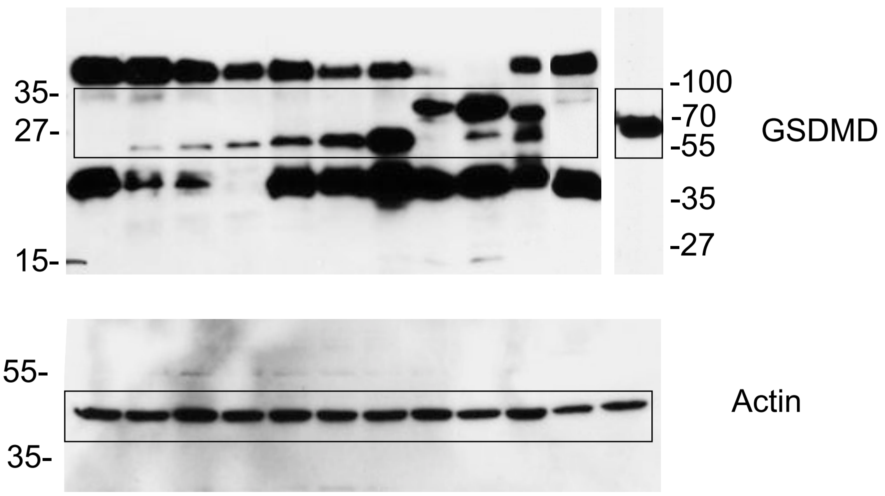

Supplementary Figure 21: Full-sized scans of Western blots in Supplementary Figure 5c, d.

Supplementary Figure 8c.

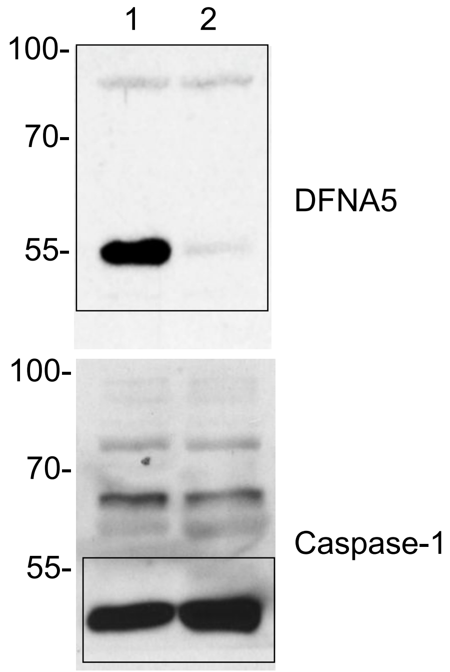

Supplementary Figure 22: Full-sized scans of Western blots in Supplementary Figure 8c.

Supplementary Figure 9b.

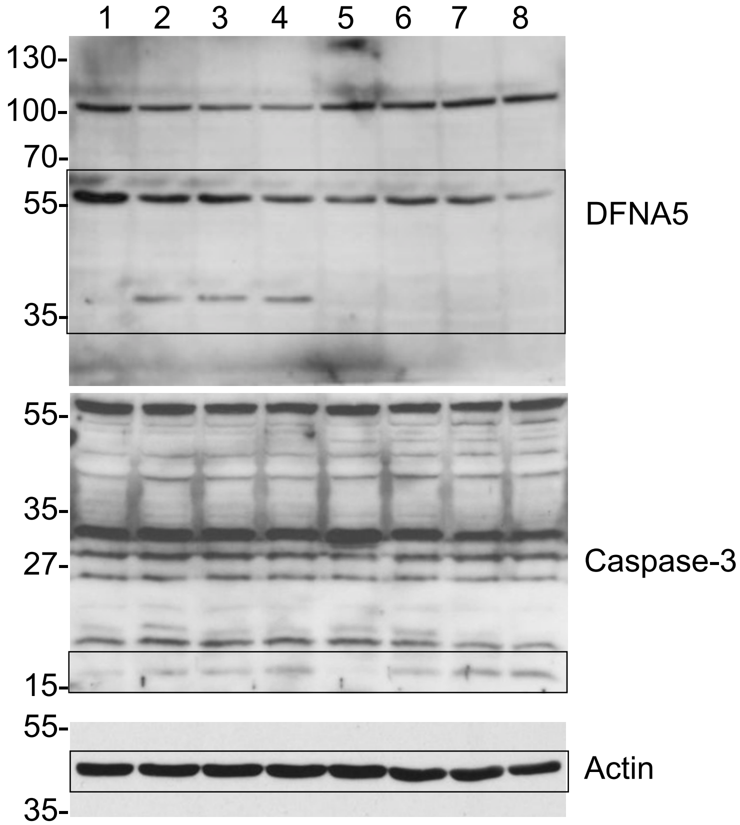

Supplementary Figure 9c.

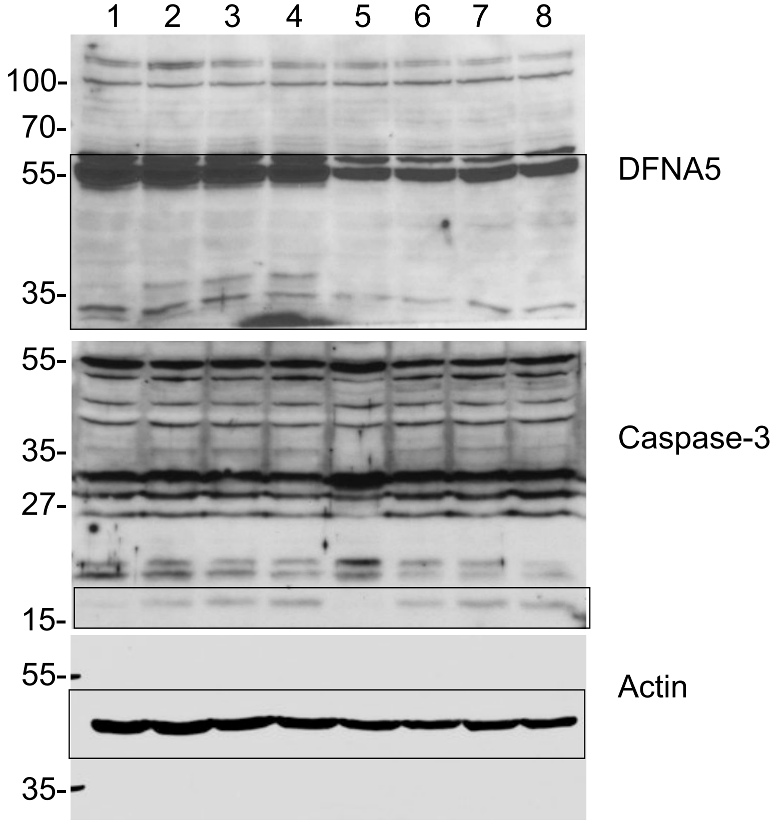

Supplementary Figure 23: Full-sized scans of Western blots in Supplementary Figure 9b, c.

Supplementary figure 10b

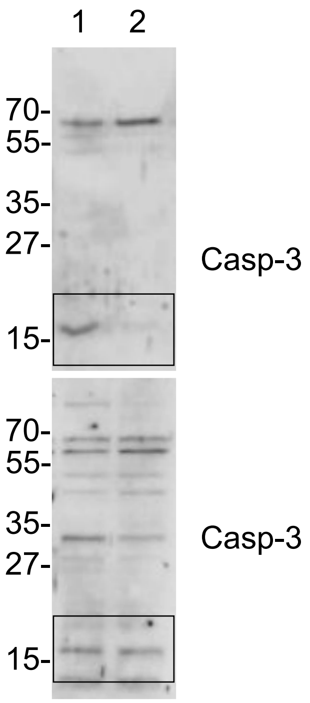

Supplementary Figure 24: Full-sized scans of Western blots in Supplementary Figure 10b.
